# Exogenous GA_3_ promotes flowering in *Paphiopedilum callosum* (Orchidaceae) through bolting and lateral flower development regulation

**DOI:** 10.1093/hr/uhac091

**Published:** 2022-04-22

**Authors:** Yuying Yin, Ji Li, Beiyi Guo, Lin Li, Guohua Ma, Kunlin Wu, Fengxi Yang, Genfa Zhu, Lin Fang, Songjun Zeng

**Affiliations:** Guangdong Provincial Key Laboratory of Applied Botany, South China Botanical Garden, Chinese Academy of Sciences, Guangzhou 510650, China; University of the Chinese Academy of Sciences, Beijing 100049, China; Guangdong Provincial Key Laboratory of Applied Botany, South China Botanical Garden, Chinese Academy of Sciences, Guangzhou 510650, China; University of the Chinese Academy of Sciences, Beijing 100049, China; Guangdong Provincial Key Laboratory of Applied Botany, South China Botanical Garden, Chinese Academy of Sciences, Guangzhou 510650, China; University of the Chinese Academy of Sciences, Beijing 100049, China; Guangdong Provincial Key Laboratory of Applied Botany, South China Botanical Garden, Chinese Academy of Sciences, Guangzhou 510650, China; Guangdong Provincial Key Laboratory of Applied Botany, South China Botanical Garden, Chinese Academy of Sciences, Guangzhou 510650, China; Guangdong Provincial Key Laboratory of Applied Botany, South China Botanical Garden, Chinese Academy of Sciences, Guangzhou 510650, China; Guangdong Key Laboratory of Ornamental Plant Germplasm Innovation and Utilization, Environmental Horticulture Research Institute, Guangdong Academy of Agricultural Sciences, Guangzhou 510640, China; Guangdong Key Laboratory of Ornamental Plant Germplasm Innovation and Utilization, Environmental Horticulture Research Institute, Guangdong Academy of Agricultural Sciences, Guangzhou 510640, China; Guangdong Provincial Key Laboratory of Applied Botany, South China Botanical Garden, Chinese Academy of Sciences, Guangzhou 510650, China; Guangdong Provincial Key Laboratory of Applied Botany, South China Botanical Garden, Chinese Academy of Sciences, Guangzhou 510650, China; Key Laboratory of South China Agricultural Plant Molecular Analysis and Gene Improvement, South China Botanical Garden, Chinese Academy of Sciences, Guangzhou 510650, China

## Abstract

*Paphiopedilum* orchids have a high ornamental value, and their flower abundance and timing are both key horticultural traits regulated by phytohormones. All one-flowered *Paphiopedilum* have additional lateral buds in the apical bract that fail to develop. In this study, an exogenous gibberellin (GA_3_) application promoted flowering of *Pathiopedilum callosum* by inducing its early bolting instead of the floral transition of dominant flowers. Applying GA_3_ effectively promoted lateral flower differentiation, resulting in a two-flowered inflorescence. GA-promoted lateral flower formation involved GA interacting with indole-3-acetic acid (IAA) and cytokinins (CTKs), given the decreased CTK content and downregulated expression of CTK synthesis genes, the increased IAA content and downregulated expression of IAA degradation, and the upregulated expression of transport genes. Further, GA acted via *PcDELLA*, *PcTCP15*, and *PcXTH9* expressed in stage 5 to promote bolting, and via expression of *PcAP3*, *PcPI*, and *PcSEP* to promote flowering. This study provides insight into mechanisms regulating flower development of *P. callosum*.

## Introduction

Plants in the *Paphiopedilum* genus are commonly known as lady’s slipper orchid because of their slipper-shaped pouch. Their flowers come in a wide range of colors and sizes. Members of this genus have a high ornamental value because of their unique flower shape and long-lasting flowering time and shelf life.

Most popular species of *Paphiopedilum* sold on the market will display one flower. All such one-flowered species have an additional flower bud in the apical bract that fails to develop further once the dominant flower is opened [1]. At present, the flowering of most *Paphiopedilum* species is non-uniform and uncontrollable. Flower abundance and timing are two key horticultural traits that substantially affect the economic and ornamental value of orchids. Therefore, by better understanding the flowering regulation mechanism, we could precisely control flowering time and increase flower productivity to greatly increase the plants’ economic value.

*Paphiopedilum* has a sympodial habit of growth. After blooming, the plant will resume growth at the base of the previous shoot. The apical meristem of each new growing shoot continually differentiates its leaves until it initiates flower buds under proper environmental conditions [2]. *Pathiopedilum callosum*, whose native range spans Thailand, Cambodia, Laos, and Vietnam [3], is an ideal material for studying the flowering of the *Paphiopedilum* genus. Firstly, this species is relatively easy to maintain in the greenhouse and it blooms uniformly. Secondly, *P. callosum* is an important parent of the *Paphiopedilum* Maudiae type, now quite a popular species on the flower market. Thirdly, under greenhouse conditions *P. callosum* usually produces a single flower, with one aborted bud in the apical bract (Supplementary Data Fig. S1); a two-flowered inflorescence is rarely observed.

Gibberellin (GA) is one of the most important plant hormones, playing a major role in floral induction and inflorescence stem elongation (bolting), in addition to anther and petal development. In the model plant *Arabidopsis*, the regulatory network of GA-mediated flower induction is comprehensively known (reviewed in [4]). A number of genes involved in GA metabolism, perception, and signaling pathways have been identified and extensively studied in many other plant species. In particular, GA20-oxidase (GA20ox) and GA3-oxidase (GA3ox) are both activating enzymes, which catalyze GA intermediates into their bioactive forms. Conversely, GA2-oxidase (GA2ox) enzymes convert bioactive GAs and their precursors into inactive forms. The genes encoding GA-activating and -deactivating enzymes regulate GA homeostasis through a feedback mechanism [5]. GA signal transduction is initiated when bioactive GA binds to its receptor GA INSENSITIVE DWARF1 (GID1) and induces a conformational change in the amino (N)-terminus of GID1; this creates a surface suitable for binding the growth repressor DELLA proteins (DELLAs), via the DELLA domain, and this subsequently leads to degradation of DELLAs by the 26S proteasome, thereby relieving their repressive effect [6, 7]. DELLAs mediate transcriptional regulation mainly through their physical interaction with transcription factors and other regulatory proteins to prevent their binding to target genes, or by acting as co-activators to promote the expression of one or more target genes [8]. In GA-mediated flowering, DELLAs and GA biosynthesis and transportation can regulate the spatial-temporal expression of floral regulatory genes, such as *FLOWERING LOCUS T* (*FT*), *LEAFY* (*LFY*), *SUPPRESSOR OF OVEREXPRESSION OF CONSTANS 1* (*SOC1*), and *SHORT VEGETATIVE PHASE* (*SVP*) [4]. Bioactive GAs are diterpene phytohormones; the most biologically active of these in plants are GA_1_, GA_3_, GA_4_, and GA_7_ [9].

Although *Paphiopedilum* orchids are prized ornamental plants, their flower-regulating mechanisms remains largely unknown because of the long vegetative growth period and huge variation in flowering among species in this genus. In this study, *P. callosum* plants were treated with a foliar application of 100 mg/l of GA_3_ to explore its role in the regulatory mechanism of flowering. To characterize this process, flowering physiology, cytological observations of floral organogenesis, major endogenous hormone profiling, and the transcriptome of floral bud development were all investigated in depth.

## Results

### Yearly cycle for a new vegetative shoot to become a flowering shoot in *P. callosum*

**Figure 1:**
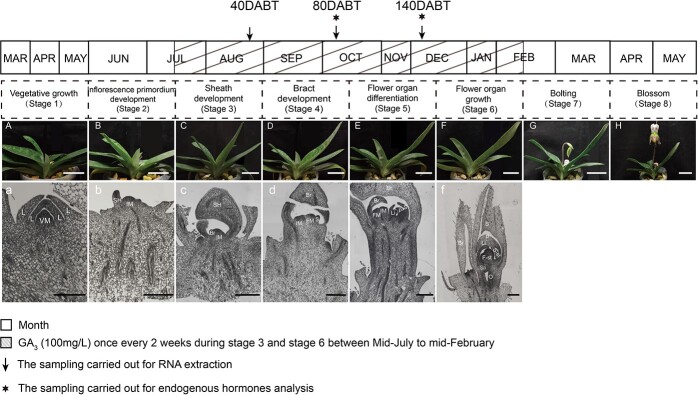
Morphology and cytology of a new shoot from vegetative to flowering in *P. callosum.* (A–H) Morphological observations of a new vegetative shoot changing into a flowering shoot at eight different developmental stages. a–f, Paraffin section observations of a new shoot at five different developmental stages. Yearly cycle from a new vegetative shoot to a flowering shoot. March–May, stage 1: a new shoot initiated from bloomed shoots (A), vegetative growth stage (a). June to mid-July, stage 2: a developing shoot with the fourth leaf (B), inflorescence primordium development stage (b). Mid-July to August, stage 3: a developing shoot with four fully developed leaves (C), sheath development stage (c). September to mid-October, stage 4: a developing shoot with the fifth leaf (D), bract development stage (d). Mid-October to mid-December, stage 5: a developing shoot with five fully developed leaves (E), flower organ differentiation stage (e). Mid-December to mid-February, stage 6: a developed shoot with swollen flower buds at the base (F), flower organs growth stage (f). Mid-February to March, stage 7: flower bud bolting from the base (G). April through May, stage 8: blooming flower (H). The *P. callosum* plants were treated with GA_3_ (100 mg/l) once every 2 weeks during stage 3 and stage 6, between mid-July to mid-February of the coming year (hatched boxes). Arrows and asterisks indicate the sampling carried out for RNA extraction and endogenous hormone analysis, respectively. L, leaf primordia; VM, vegetative meristem; SH, sheath; IM, inflorescence primordium; Br, bract; FM, floret primordium; S, sepal; P, petal; Li, lip; S-st, sterile stamen; F-st, fertile stamen; Sti, stigma; O, ovary. Scale bars: A–H = 5 cm, a–h = 500 μm.

Adults of *P. callosum* usually bloom from April to May each year under our greenhouse conditions. These flowering plants usually have five leaves and one inflorescence sheath. The development of a new vegetative shoot into a flowering shoot of *P. callosum* can be divided into eight consecutive stages (Fig. 1). Stage 1: In March, a new shoot capable of flower differentiation grows outward from the base part of the tissue that has already flowered (Fig. 1A). Before June, this new emerging shoot is vegetative, having a narrow shoot apical meristem with leaf primordia on its flanks. These meristem cells harbor a large nucleus and are small and closely packed together (Fig. 1a). Stage 2: At the onset of June, the fourth leaf begins to elongate and grow (Fig. 1B). The shoot apical meristem then widens and becomes enlarged, and the inflorescence sheath primordium emerges from it (Fig. 1b). Stage 3: By the end of August, this new shoot now has four fully mature leaves (Fig. 1C). The inflorescence sheath primordium folds in half to form a protective sheath-like structure; due to the non-uniform cell division and expansion, the bract primordium emerges (Fig. 1c). Stage 4: During September to mid-October the fifth leaf begins to grow (Fig. 1D). The bract primordium continues to grow, and its formation is similar to that of the inflorescence sheath primordium. Non-uniform cell division and expansion also result in a protective sheath-like structure forming. The apical meristem in the bract continues to grow and expand, forming two semicircular protrusions in the middle, corresponding to the floret primordia. One floret primordium differentiates faster than the other, and of these the sepal primordium differentiates first; this is referred to as the dominant flower, while the remainder are lateral flowers (Fig. 1d). Stage 5: From mid-October to mid-December, accompanied by continuous elongation of the fifth leaf, the dominant flower primordium continues to grow, and a gap is formed. Later, this gap further differentiates to form two new protrusions: a petal primordium and lip primordium. Meanwhile, the inflorescence axis also undergoes pronounced elongation. Note that each floret primordium is covered with a single bract (Fig. 1E, e). Stage 6: From mid-December to mid-February of the following year, the differentiated flower organs grow and develop further; flower volume continues to increase, with buds now clearly visible at the base. The new shoot has five fully mature leaves (Fig. 1F) and dominant flower organs are fully developed. The sepal, petal, and lip primordia continue to enlarge, with the fertile stamen and sterile stamen then developing inside the petal primordium. The fertile stamen primordium is disk-like, while the sterile stamen primordium is tubular-like in shape. The stigma and ovary are formed on the inner side of the fertile stamen and sterile stamen. Most lateral floral buds fail to develop further once dominant floral organogenesis is completed (Fig. 1f). Stage 7: From mid-February to March, rapid elongation of the stem internodes occurs, a process called bolting. The dominant flower gradually increases in size and opens the outermost bracts (Fig. 1G). Stage 8: Eventually, the inflorescence expands, followed by opening of the dominant flower (Fig. 1H).

### Exogenous GA_3_ application promotes flowering by inducing early bolting and increasing the number of flowers

The *P. callosum* plants were treated with GA_3_ (100 mg/l) once every 2 weeks during stage 3 and stage 6, between mid-July and mid-February (hatched boxes in Fig. 1). This exogenous GA_3_ treatment can effectively promote early flowering, in that the bolting and flowering dates were respectively 33 and 35 days earlier than in the control group. Moreover, the number of flowers per plant increased by 67.60%. The exogenous GA_3_ treatment significantly promoted elongation of the inflorescence stem and caused the pedicel to bend; the proportion of plants with a curved inflorescence reached 63.33%. In terms of flower size, no significant differences were detected with respect to petal, lip, and sepal size (Table 1).

**Figure 2:**
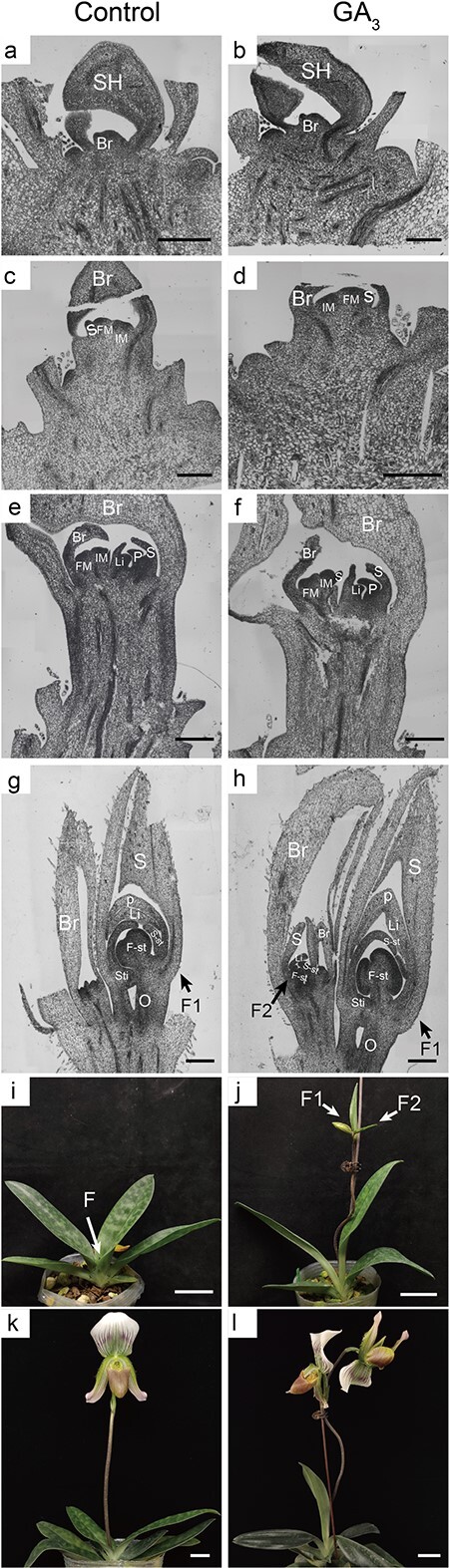
Exogenous GA_3_ treatment can initiate lateral flower differentiation, resulting in the two-flowered inflorescence. Paraffin sections of buds collected from the control and GA_3_-treated group at 40 DABT, both in the sheath development stage (stage 3) (a, b); at 80 DABT, both in the bract development stage (stage 4) (c, d); at 140 DABT, both in the flower organ differentiation stage (stage 5) (e, f); at 180 DABT, both in flower organs growth stage (stage 6), where the lateral flower of the control group was stagnant (g) while that of the GA_3_ group had developed as the dominant flower (h). Shown in (i) is a plant from the control group at 220 DABT in stage 6; in (j) a plant from the GA_3_-treated group at 220 DABT in the bolting stage (stage 7); in (k) a blossom flower from control group at 280 DABT, with one flower; and in (l) a blossom flower from GA_3_-treated group at 250 DABT, with two flowers. SH, sheath; IM, inflorescence primordium; Br, bract; FM, floret primordium; S, sepal; P, petal; Li, lip; S-st, sterile stamen; F-st, fertile stamen; Sti, stigma; O, ovary; F, flower bud; F1, dominant flower; F2, lateral flower. Scale bars: A–D, G, H = 500 μm; E, F = 5000 μm; I, J = 5 cm; K, L = 2.5 cm.

To get detailed information on how GA_3_ affected floral development, we made cytological observations of the control and GA_3_-treated buds (Fig. 2). In control buds, at 40 days after the beginning of treatment (DABT), the apical meristem was wrapped by the inflorescence sheath and the flower bud was in stage 3 (Fig. 2a). The meristem emerged, became enlarged, and then differentiated into a floret primordium belonging to stage 4 (80 DABT; Fig. 2c). The meristem then entered stage 5, at which point floral organs began to differentiate. The sepals, petals, and lip were visible in the dominant flower (140 DABT; Fig. 2e), and they grew more and developed further, such that the fertile stamen and sterile stamen were discernible, corresponding to stage 6 (180 DABT; Fig. 2g). Later, the flower buds continued to swell, and flower buds were clearly visible at the base. These flower buds remained in stage 6, however (220 DABT; Fig. 2i). By 280 DABT, the dominant flower had bloomed and there was an aborted bud in the apical bract (Fig. 2k). For GA_3_-treated plants (Fig. 2b, d, f, h, j, k), their morphology was not significantly different from the control group at 40, 80, and 140 DABT; however, at 180 DABT the GA_3_ treatment did initiate lateral flower differentiation, so that the development of the lateral flower soon followed that of the dominant flower. Then GA_3_-treated plants bolted quickly and bloomed in advance, forming a two-flowered inflorescence, resulting in a higher flower abundance per plant. These observations clearly demonstrated that GA_3_ is capable of inducing lateral flower differentiation and promoting early flowering by advancing the onset of bolting.

**Figure 3:**
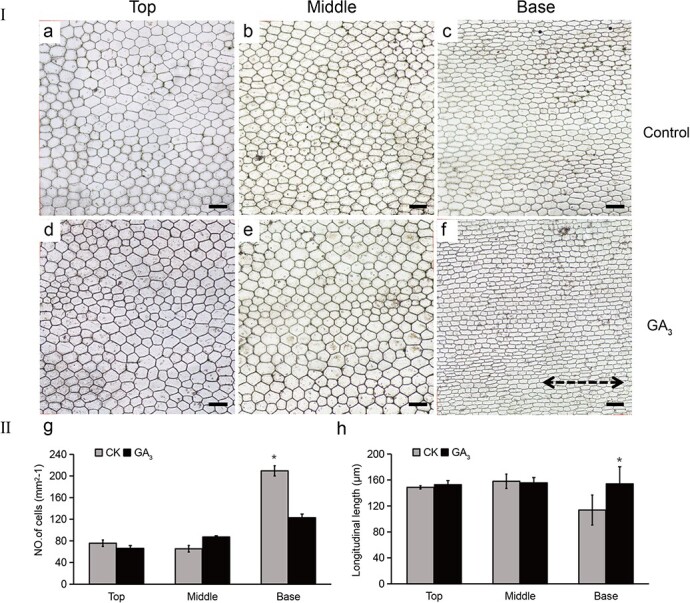
Exogenous GA_3_ promotes cell elongation and an increase in the numbers of cells in the base of *P. callosum* leaves. I. Abaxial epidermal cells in the top, middle, and basal regions of the top-first leaf in stage 6 were observed under an optical microscope. (a–c) Control; (d–f) treated with GA_3_. Scale bar = 100 μm. The dashed two-headed arrow indicates the longitudinal direction. II. Cell number (g) and length (h) of the top, middle, and basal regions. Values are mean ± standard error. An asterisk indicates a significant difference from the control (Student’s *t*-test, *P* < .05).

The GA_3_ treatment also significantly promoted leaf elongation. Mature *P. callosum* plants generally have five leaves: these are termed the first, second, third, fourth, and fifth (from top to bottom). The first, second, and third are larger functional leaves, whereas the smaller fourth and fifth leaves mainly protect the tiller buds at their early stage and gradually turn yellow and peel off as the plant grows and develops. When the plant enters stage 6 all leaves are fully developed. Compared with the control, the first and second leaves of GA_3_-treated plants increased significantly in length, by 62.05 and 35.12%, respectively (Table 2). Applying GA_3_ significantly affected both cell size and density. Observing the first leaf’s abaxial epidermal cellular morphology in stage 6 in the top, middle, and basal regions revealed that the number of cells after treatment with GA_3_ only increased in the basal leaf parts, whereas the other two parts barely differed. Nevertheless, the basal cells had the greatest longitudinal length after the GA_3_ treatment (Fig. 3).

### Effect of GA_3_ treatment on levels of endogenous hormones in flower buds

**Figure 4:**
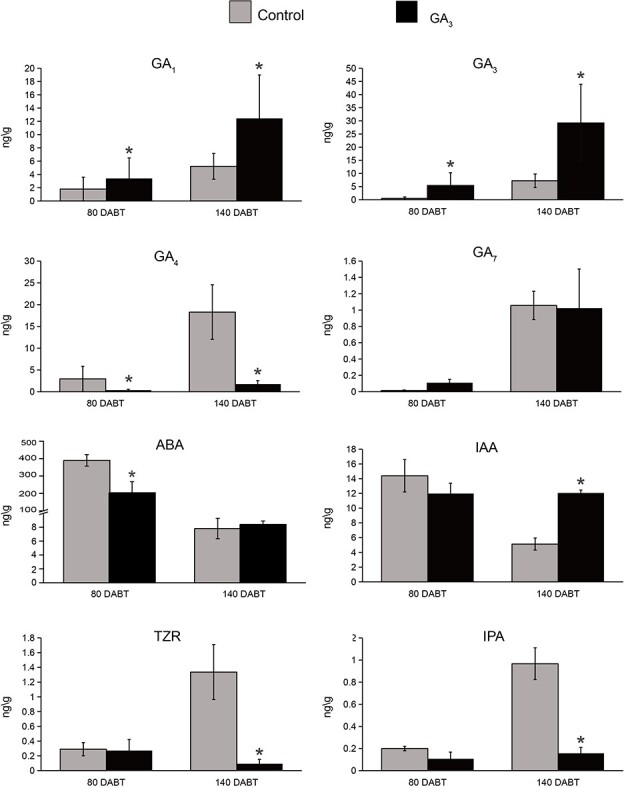
Levels of endogenous hormones GAs, ABA, IAA, TZR, and IPA at 80 and 140 DABT in GA_3_-treated *P. callosum* and the control group. Flower buds (100 mg) were freshly harvested at 80 and 140 DABT. Data represent the average of three biological replicates. Values are mean ± standard error. Hormone level is indicated as ng/g fresh weight (FW). An asterisk indicates a significant difference from the control group (Student’s *t*-test, *P* < .05)

To investigate the dynamic hormonal changes of *P. callosum* plants in response to exogenous GA_3_, the endogenous hormone levels in the buds were measured at two stages: 80 and 140 DABT. Exogenous GA_3_ treatment increased the endogenous GA_1_ content by 88.50% (80 DABT) and 138.81% (140 DABT), and the endogenous GA_3_ content by 9-fold (80 DABT) and 3-fold (140 DABT), whereas it reduced the endogenous GA_4_ content by 7-fold (80 DABT) and 9-fold (140 DABT). However, for GA_7_ there was no significant difference vis-à-vis the control at either stage (Fig. 4).

At 80 DABT, based on cytological observations, the buds collected from the control and GA_3_ groups were in the bract development stage (stage 4). Exogenous GA_3_ not only caused significant changes in endogenous bioactive GAs but also a significant decrease in the abscisic acid (ABA) content of flower buds, yet it did not significantly influence their indole-3-acetic acid (IAA), *trans*-zeatin riboside (TZR), or isopentenyl adenosine (IPA) content. At 140 DABT, buds collected from both the control and the GA_3_ group were in the flower organ differentiation stage (stage 5) according to cytological observations. The ABA content was similar between GA_3_-treated and control plants; however, compared with the bract development stage, it had decreased significantly from 390.23 to 7.81 ng/g and from 204.01 to 8.39 ng/g, respectively. This result indicated the reduction in ABA levels could be related to the later stage of floral organ growth and development. Furthermore, in stage 5 the IAA content of GA_3_-treated plants significantly exceeded that of the control, and vice versa for cytokinins (CTKs). To determine the CTK level in *P. callosum*, TZR, IPA, zeatin and isopentenyladenine (iP) were all quantified; all but the last were significantly reduced in GA_3_-treated plants (Supplementary Data Fig. S2).

We also examined the levels of other hormones, namely salicylic acid (SA) and jasmonic acid (JA). After GA_3_ treatment, from 80 to 140 DABT, the SA level decreased but the JA level increased (Supplementary Data Fig. S2).

### Global mRNA expression signatures of GA_3_-treated flower buds in different development stages

Clean data (127.29 Gb in total) were obtained from 18 libraries (2 treatments × 3 biological replicates × 3 developmental stages). According to the paraffin sectioning results, flower buds of either the control or GA_3_-treated group collected at 40, 80, and 140 DABT were in stage 3, 4, and 5, respectively (Fig. 2). Hence, we named these two sets of samples CK3–5 and GA3–5. Overall, the Q30 of all clean reads was >91%, with a GC content of 46.66–52.37. Detailed statistics of the clean reads are listed in Supplementary Data Table S1. Lacking a reference genome sequence of *P. callosum*, we *de novo* assembled the total clean reads, thus obtaining 157 463 unigenes having an average length and N50 length of 811.51 and 1282 bp, respectively (Supplementary Data Table S2). Principal component analysis (PCA) was performed for all samples spanning the flower developmental stages (Supplementary Data Fig. S3a). Except for stage 3, the other samples from different biological replicates clustered separately in the PCA biplot, following their distinct developmental stages.

A total of 157 463 unigenes were searched against six publicly available databases—NCBI non-redundant protein sequences (NR), Kyoto Encyclopedia of Genes and Genomes (KEGG), the Clusters of Orthologous Groups (COG), Gene Ontology (GO), Protein family (Pfam), and Swiss-Prot—by using the BLASTX tool. This resulted in a total of 69 997 (44.45%) unigenes with at least one putative function annotated in one database (Supplementary Data Table S3). Sequence comparisons revealed that *P. callosum* transcripts were highly similar to those of *Dendrobium catenatum* (37.68%), *Apostasia shenzhenica* (7.20%), and *Quercus suber* (6.69%) (Supplementary Data Fig. S3b).

### Differential gene expression and KEGG enrichment analysis

**Figure 5:**
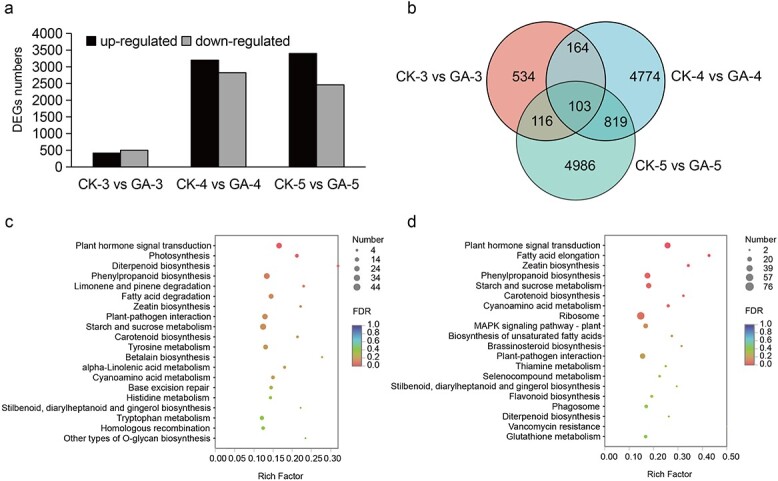
Analysis of DEGs from samples at three different flower developmental stages of *P. callosum.* (a) Histogram of the number of up-/downregulated DEGs in each comparison group. (b) Venn diagram of all DEGs. (c) KEGG enrichment analysis of the DEGs in stage 4 between GA_3_-treated and control plants. (d) KEGG enrichment analysis of the DEGs in stage 5 between GA_3_-treated and control plants.

A total of 12 801 differentially expressed genes (DEGs) were identified between the GA_3_-treated and control plants in the three different comparison groups (i.e. CK-3 versus GA-3, CK-4 versus GA-4, and CK-5 versus GA-5). That of CK-3 versus GA-3 had the fewest DEGs, with 917, of which 416 were upregulated and 501 were downregulated. For the CK-4 versus GA-4 grouping, the number of DEGs was 6024, with 3200 and 2824 of these upregulated and downregulated, respectively. For CK-5 versus GA-5, there were 5860 DEGs, with 3400 upregulated and 2460 downregulated (Fig. 5a). These results suggested that, among the three stages, the GA_3_ treatment affected gene expression mostly at the bract development stage and floral organ differentiation stage. A total of 103 DEGs were distributed among the pairwise comparisons of all three developmental stages; hence, they could be involved in flower development (Fig. 5b).

We also conducted a KEGG enrichment analysis for DEGs in CK-4 versus GA-4 and in CK-5 versus GA-5 (Fig. 5c and d). For CK-4 versus GA-4, plant hormone signal transduction, photosynthesis, diterpenoid biosynthesis, and phenylpropanoid biosynthesis were the top-enriched KEGG pathways, suggesting their involvement in the morphological differentiation of floret primordia. For CK-5 versus GA-5, which represent the key stage of floral organ growth, analysis of the DEGs showed that pathways for plant hormone signal transduction pathway, starch and sucrose metabolism, phenylpropanoid biosynthesis, zeatin biosynthesis, and fatty acid elongation were all highly enriched. Of them, the first pathway was highly enriched throughout the three flower developmental stages, implying that the dynamic changes in hormones are closely related to flower development in *P. callosum*.

### Differentially expressed genes related to hormone metabolism and transduction

**Figure 6:**
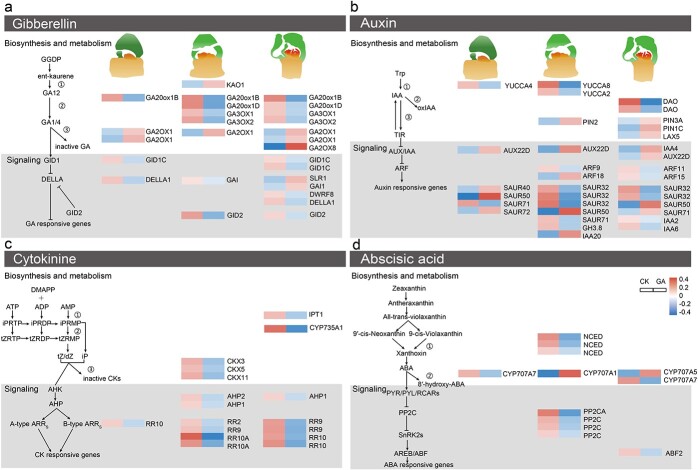
Heat map of DEGs related to plant hormone metabolism and signaling. Following the GA_3_ treatment in three different flower developmental stages of *P. callosum*: (a) DEGs with roles in GA biosynthesis, deactivation, and signaling pathways; (b) DEGs with roles in IAA biosynthesis, deactivation, and signaling pathways; (c) DEG_S_ with roles in CK biosynthesis, deactivation, and signaling pathways; (d) DEGs with roles in ABA biosynthesis, deactivation, and signaling pathways. Blue squares indicate downregulation, whereas red squares indicate upregulation. The color scale corresponds to the average log_10_ (FPKM+1; FPKM, fragments per kilobase of transcript per million mapped reads) and *Z*-score value (normalized by R software).

In the GA biosynthesis pathway, *ent*-kaurenoic acid oxidase (KAO), GA20ox, and GA3ox are the main enzymes that synthesize GAs. *KAO* is an early GA biosynthesis gene, mainly responsible for oxidizing *ent*-kaurenoic acid, *ent*-7-hydroxykaurenoic acid, and GA_12_-aldehyde to GA_12_. Both *GA20ox* and *GA3ox* are soluble 2-oxoglutarate-dependent dioxygenases (2ODDs) in the later stage of GA biosynthesis, which, via two parallel pathways, convert GA_12_ and GA_53_ into various GA intermediates and bioactive GAs [10]. We found a total of five *GA20ox* genes and four *GA3ox* genes in the three flower developmental stages; all were downregulated in response to the GA_3_ treatment. Furthermore, *KAO* was upregulated in stage 4 after the GA_3_ treatment (Fig. 6a; Supplementary Data Table S4). GA2ox is a major GA deactivation enzyme that catalyzes 2β-hydroxylation to irreversibly inactivate GAs, thereby controlling the concentration of bioactive GAs [9]. In GA_3_-treated buds, six *GA2ox* genes were upregulated at the three developmental flower bud stages spanning sheath development to floral organ differentiation. The feedback regulatory mechanism by which GA maintains GA homeostasis works by inhibiting the expression of *GA20ox* and *GA3ox* while promoting the expression of *GA2ox*, to control the concentration of active GA after exogenous GA_3_ application. As central GA-signaling components, the GA receptor *GID1*, DELLA proteins, and the Skp1-Cullin-F-box (SCF) E3 ubiquitin-ligase complex, via the F-box protein [SLEEPY1 (SLY1)] in *Arabidopsis* or GIBBERELLIN INSENSITIVE DWARF2 (GID2) in rice, also function in GA homeostatic regulation [8]. Here, *GID1* and *GID2* were downregulated during all three stages; similarly, two genes encoding DELLA proteins exhibited downregulation following the GA_3_ treatment in both stage 3 and stage 4. By contrast, in stage 5, of the four genes encoding DELLA proteins, two showed upregulation and two showed downregulation in response to the GA_3_ treatment. These results indicated that exogenous GA_3_ application modified the expression of genes involved in biosynthesis, degradation, and signal transduction pathways.

Besides GA, auxin, ABA, and CTK also play pivotal roles in flower development [11–13]. In the biosynthesis of IAA, the YUCCA family of flavin monooxygenases convert IPA to IAA [14, 15]. Two *YUCCA* genes were downregulated during stage 4, in tandem with the relatively low level of IAA characterizing that stage. DIOXYGENASE OF AUXIN OXIDATION (DAO) proteins belong to the 2-oxoglutarate (2OG) Fe(II) oxygenase family, which is responsible for catalyzing the conversion of active IAA into biologically inactive 2-oxoindole-3-acetic acid (OxIAA) and is essential for ensuring auxin homeostasis [16]. The PIN-FORMED (PIN) proteins and AUXIN1/LIKE-AUX1 (AUX1/LAX) proteins are both important auxin transporters in plants. PIN proteins efflux auxin and establish auxin gradients, and AUX1/LAX proteins act to influx auxin from the apoplast into the cytoplasm [17, 18]. Here, during stage 5, two *DAO* genes were downregulated, while two *PIN*s and one *LAX5* were upregulated, a pattern consistent with the significantly high IAA level during this stage. The signaling pathway downstream of IAA includes the *auxin response factor* (*ARF*), *Small Auxin-Up RNA* (*SAUR*), *AUXIN/INDOLE-3ACETIC ACID* (*Aux/IAA*), and *Gretchen Hagen3* (*GH3*) genes [18]. A total of 25 downstream auxin-responsive DEGs were found in the three flower developmental stages, whose expression profiles were complex (Fig. 6b; Supplementary Data Table S4).

CTKs can mediate cell proliferation by affecting cell division and/or cell differentiation. In the metabolic pathways of CTKs, gene expression levels of *adenylate isopentenyltransferase* (*IPT*) and *cytochrome P450 monooxygenase* (*CYP735A*), encoding crucial enzymes for the synthesis of CTKs, were both downregulated during stage 5. Response regulators (ARRs) serve as the major downstream component of the cytokine signal pathway [19]. A total of nine type-A ARRs were annotated in the three flower development stages, and all of them were downregulated in the GA_3_-treated group of *P. callosum* plants (Fig. 6c; Supplementary Data Table S4).

The hormonal action of ABA is precisely controlled, by balancing its biosynthesis and catabolism. Three DEGs associated with the ABA biosynthetic pathway, and one DEG in the ABA catabolism pathway, were discovered in stage 4. Hence, combined with the changed endogenous ABA level occurring in this stage, it is plausible that the higher expression of ABA *8′-hydroxylase* (*CYP707A*) and lower expression of *9-cis-epoxycarotenoid dioxygenase* (*NCED*) contributed to the reduced ABA levels (Fig. 6d; Supplementary Data Table S4).

### Differentially expressed genes related to flowering and bolting

**Figure 7:**
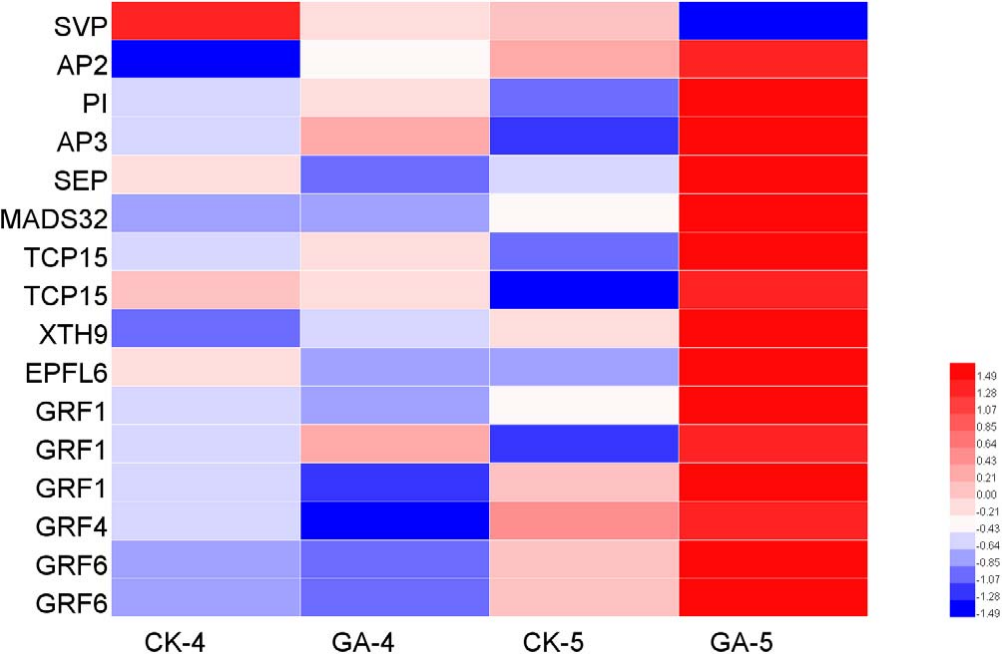
Heat map of DEGs related to flowering and bolting. The blue squares indicate downregulation, whereas the red squares indicate upregulation. At the bottom is the color scale corresponding to the average log_10_ (FPKM+1; FPKM, fragments per kilobase of transcript per million mapped reads) and *Z*-score value (normalized by R software).

At stages 4 and 5, a total of 39 flowering-related DEGs were identified between the GA_3_ treatment group and the control: namely, the photoperiodic flowering-related genes *CONSTANS-LIKE* (*COL*), *CYCLING DOF FACTOR* (*CDF*), *GIGANTEA* (*GI*), *CRYPTOCHROME* (*CRY*), and *FD*; the temperature-sensitive flowering pathway genes *FRIGIDA* (*FRI*) and *SHORT VEGETATIVE PHASE* (*SVP*); and the sugar pathway gene *SUCROSE SYNTHASE* (*SUS*), in addition to other transcription factors, such as SQUAMOSA PROMOTER BINDING PROTEIN-LIKE (SPLs) and NUCLEAR FACTOR Y SUBUNIT A/B (NF-YA/Bs), as well as other MADS-box family transcription factors. As seen in Supplementary Data Table S5, the MADS-box transcription factor SVP, which represses floral transition during the vegetative phase by regulating several floral pathway integrators, including *FT* and *SOC1*, was significantly downregulated after the GA_3_ treatment during stages 4 and 5. The other MADS-box genes *APETALA3* (*AP3*)*, PISTILLATA* (*PI*), *SEPALLATA* (*SEP*), and *MADS32* and the AP2/ERF transcription factor family gene *APETALA2* (*AP2*) are floral organ identity genes which contribute to floral organogenesis. All these genes were significantly upregulated in stage 5 after treating *P. callosum* with GA_3_ (Fig. 7).

Inflorescence stem elongation (bolting) is another pivotal event that occurs during the reproductive development of *P. callosum*. Stem elongation includes two continuous processes: cell division and cell elongation. GA not only induces the expression of cell-cycle regulatory proteins to promote cell division but also promotes cell elongation by increasing the extensibility of the cell wall. Regarding cell division, in *Arabidopsis*, DELLA proteins can downregulate the expression of several important cell-cycle genes and constrain cell division by directly repressing class I TCP transcription factors [20, 21]. Here, the gene *TCP15*, which encodes a non-canonical basic helix–loop–helix (bHLH) transcription factor involved in regulating cell proliferation, was upregulated in stage 5 in the GA_3_-treated group. In terms of cell elongation, XTH9, a member of the XTH family (xyloglucan endotransglucosylase/hydrolases), can disrupt the polysaccharide structure, thus leading to a loosened cell wall; GA signaling induces transcription of *XTH9* by increasing cell elongation of the inflorescence stem in *Arabidopsis* [22]. In this respect, the *XTH9* gene was significantly upregulated in stage 5 in the GA_3_-treated group in our study. GRF1 (GROWTH-REGULATING FACTOR1) can play a regulatory role in GA-induced stem elongation in *Oryza sativa* [23], while EPIDERMAL PATTERNING FACTOR-LIKEs (EPFLs) are known to be involved in the coordinated growth of inflorescence architecture in *Arabidopsis* [24]. Specifically, we found that six *GRF1*s and one *EPFL6* gene were all upregulated in stage 5 in the GA_3_-treated group (Fig. 7).

### Quantitative real-time–PCR verification

**Figure 8:**
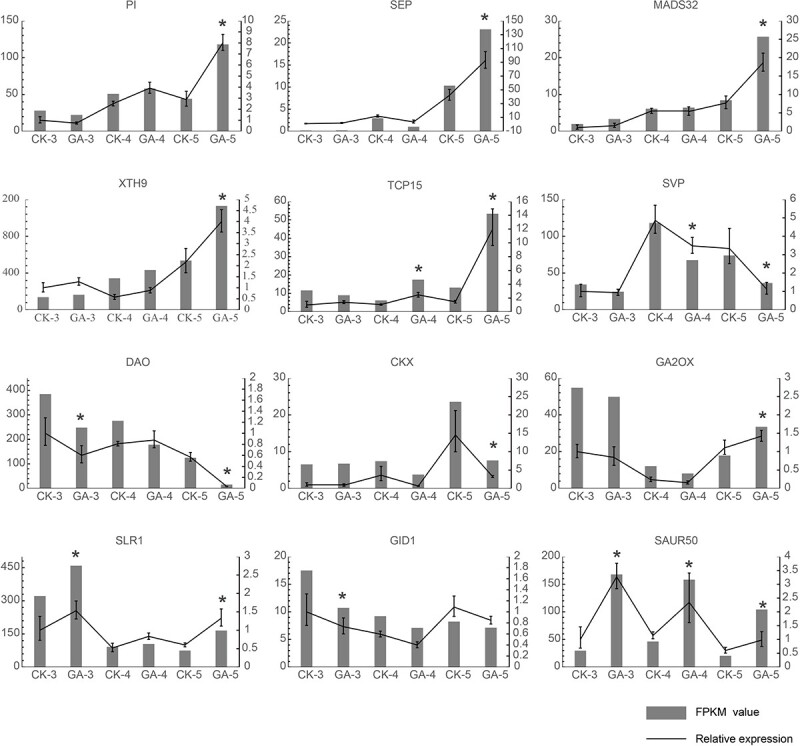
Validation of gene expression levels in the GA_3_-treated group and control at the three stages of flower development by qRT–PCR. The reference gene was 60s (TRINITY_DN24966_c0_g2). The samples used for the RNA extraction came from three biological replicates. On the *x*-axis is the number of days since GA_3_ treatment application when the buds were harvested (likewise in the control). On the left *y*-axis are the RPKM (reads per kilobase per million reads) values; on right *y*-axis are the relative expression levels. Error bars are the standard deviation of the mean (*n* = 3). An asterisk indicates a significant difference from the control (Student’s *t*-test, *P* < .05).

A total of 12 genes, mainly involved in bolting, flower development, and plant metabolism hormone signaling (obtained from the three development stages and two treatments), were selected for quantitative real-time polymerase chain reaction (qRT–PCR) to confirm the accuracy and reproducibility of the RNA-Seq results. Consistent with the transcriptome data, expression levels of the MADS-box genes *PI*, *SEP1*, and *MADS32* and the bolting-related genes *XTH9* and *TCP15* were all lower in stages 3 and 4, yet similar between the two treatment groups. In stage 5, their expression levels increased significantly, being significantly higher in the GA_3_-treated group than the control. The MADS-box gene *SVP* had a lower expression level in stage 3, which was similar between the GA_3_-treated group and control, but then its expression became significantly higher and different between them in stages 4 and 5 (Fig. 8). In stage 5, the genes *DAO* and *CKX* for plant metabolism hormone signaling displayed relatively high transcript levels in the control, but low transcript levels under the GA_3_ treatment, and vice versa for *GA2ox*. Genes for the hormone signal transduction DELLA protein, along with *GID1* and *SAUR50*, differed in their expression across the three development stages and also between the two treatments. Expression levels of each gene as determined by qRT–PCR were consistent with the prior RNA-Seq results.

## Discussion

### Growth habit and characteristics of *P. callosum*

According to their growth habit, orchids may be divided into two broad groups. Monopodial orchids, such as *Phalaenopsis*, have an apical meristem that remains active indefinitely while the bud primordia, which are capable of developing inflorescences, are formed in the axil of each leaf. Two or three inflorescences can be expected from the leaf axil under a 6-benzylaminopurine (6-BA) hormone treatment [25]. In sympodial orchids, such as *Paphiopedilum*, a new shoot may arise from the base of the previous shoot and differentiation of the flower bud primordium is directly associated with every new shoot [2]. Unlike *Phalaenopsis*, *Paphiopedilum* plants have only one terminal bud primordium, which produces a single inflorescence. According to their number of flowers, members of the *Paphiopedilum* genus can be divided into one-flowered species, such as *P. purpuratum*, *P. venustum*, and *P. helenae*, or multi-flowered species, such as *P. stonei*, *P. sanderianum*, and *P. victoria-regina.* Interestingly, both one-flowered and multi-flowered species possess more than one floral bud (Supplementary Data Fig. S4), suggesting they all harbor the potential to bloom multiple flowers.

The orchid *P. callosum* is a typical single-flowered *Paphiopedilum*, and through its paraffin sectioning we were able to explore the whole development process of *P. callosum* buds, from vegetative to reproductive growth (Fig. 1). In stage 5, the dominant floral organogenesis is complete, and the lateral ones begin to initiate. Stage 5 is therefore the key stage for the regulation of flower numbers: in the absence of an intervention before this stage, the lateral buds will become aborted buds attached to the dominant flower. The ensuing flower count may depend, however, to a great extent on the environmental factors at the time of flower-bud initiation. Indeed, the mechanism that controls the number of flowers in this species is currently unclear.

### Exogenous GA_3_ application promotes early flowering and increases the number of flowers

GA is among the most important phytohormones functioning in flower induction, and an exogenous GA_3_ application is commonly used to study the flowering mechanisms of various plants. For example, GA_3_ can promote the flowering of the short-day plants chrysanthemum [26] and lily [27], but inhibits the flowering of woody plants, such as apple [28] and citrus [29]. For orchid plants, the impact of GA_3_ varies among species. For example, applied alone, exogenous GA_3_ negligibly affected flower induction in *Phalaenopsis* [25]. Nevertheless, an injection of optimum levels of GA_3_ can break flower bud dormancy under high temperature and promote the continuous development of inflorescences [30, 31]. By itself, a GA_3_ application cannot promote flower bud differentiation in *Dendrobium*, but applying GA_3_ can significantly augment the promoting effect of 6-BA on flower bud differentiation [32]. In other work, an exogenous GA_3_ treatment was shown to induce the flowering of *Miltoniopsis* orchids and a *Cattleya* hybrid [33, 34]. In our study, 100 mg/l of exogenous GA_3_ was sprayed on the flowering shoot of *P. callosum* plants once every 2 weeks, to explore the role of GA in the flowering process of *Paphiopedilum*. Flower physiology observations confirmed that the GA_3_ treatment effectively advanced the onset of flowering, in that the bolting and flowering dates were respectively 33 and 35 days earlier than in the non-GA_3_-treated group (control). The early-flowering phenotype was consistent with other reports in *Paphiopedilum* [35, 36]. In *Arabidopsis*, GA is crucial for flower initiation because it induces a switch from vegetative growth to flowering under short photoperiods [37]. Our interpretation differs from that, however, when considering the detailed cytological observations (Fig. 2) and gene expression results (Fig. 5) in this study. No significant morphological differences were discernible before stage 5. Furthermore, the analysis of flowering-related genes indicated that the GA treatment affected floral development mostly at the bract development stage and floral organ differentiation stage. These results suggest that an exogenous GA_3_ treatment was incapable of driving the vegetative apical meristem to become an inflorescence meristem for organogenesis of the dominant flower. Instead, the early-flowering phenotype was formed by cell elongation of the flowering stalk that was induced by the GA_3_ treatment (Fig. 2i and j). Flowering abundance was also effectively regulated by the GA_3_ treatment, as the number of flowers per plant increased by 67.60% relative to the control (Table 1). The lateral floral buds failed to develop further once the dominant flower had bloomed. One plausible explanation for this is that the successful development of a dominant flower somehow inhibits the formation of lateral flowers. Based on the cytological observations, we speculated that GA_3_ treatment could effectively mitigate inhibition of lateral flower development.

**Table 1:** Effect of exogenous GA_3_ foliar spraying on physiological and morphological characteristics of *P. callosum^a^*.

	Percentage	Flower	Mean days from start of treatment to		Curved	Petal		Lip		Sepal		Inflorescence
Treatment	flowering	no. per plant			inflorescence							length
	(%)		Bolting	Flowering	rate (%)	Length	Width	Length	Width	Length	Width	(cm)
						(cm)	(cm)	(cm)	(cm)	(cm)	(cm)	
Control	64.86	1.08±0.06	248.78±0.55	279.44±0.90	0.00	5.62±0.16	1.74±0.12	4.51±0.11	2.74±0.07	4.92±0.14	5.26±0.12	14.96±0.51
GA_3_	70.00	1.81±0.09^*^	215.33±0.65^*^	244.56±0.63^*^	63.33^*^	5.72±0.10	1.83±0.10	4.34±0.11	2.64±0.15	5.16±0.26	5.16±0.28	19.74±0.98^*^

a The control group consisted of plants sprayed with 5 ml of distilled water containing an equivalent amount of ethanol. Spraying was carried out once every 2 weeks during stage 3 to stage 6, with a hand sprayer.

^*^Significant difference from the control (Student’s *t*-test, *P* < .05). Values are mean ± standard error.

### Effects of GA_3_ on endogenous hormone content related to the lateral flower formation

An exogenous GA_3_ application stimulated the synthesis of endogenous GA_3_ and GA_1_, yet the level of endogenous GA_4_ was reduced in *P. callosum*. Exogenous GA_3_ treatment may have led to downregulation of upstream biosynthesis genes, such as *GA20ox* and *GA3ox*, resulting in a decrease in GA_4_ synthesis to maintain the overall GA level in plants. The GA_1_ content increased because the extra GA_3_ can be converted to GA_1_ in the process [9]. Greater endogenous GA_3_ and GA_1_ contents could therefore be involved in lateral flower differentiation and later flower development in *P. callosum*. In *Arabidopsis*, rising GA levels were able to suppress the reversion of the floral meristem to an inflorescence shoot apical meristem in the mutants *apetala1* (*ap1*) and *apetala2* (*ap2*), thus demonstrating the importance of GA in the maintenance of floral meristem identity [38, 39]. Likewise, in *Phalaenopsis* orchids, continued inflorescence development and flower bud initiation were closely associated with increased levels of endogenous GAs [31]. Besides the greater content of endogenous GA, exogenous GA_3_ also elicited significant changes in the content of endogenous IAA and CTKs at 140 DABT (stage 5). Generally, CTKs play an important role in regulating the development of apical meristems by influencing stem cell fate at the shoot apical meristem [40]. Auxin is another key hormone that regulates formation and differentiation of the shoot apical meristem. The inflorescence meristem of the *Arabidopsis pin-formedl-1* (*pin1*) mutant does not produce flowers, but the application of auxin triggers flower organogenesis in its shoot, showing that auxin is required for and sufficient to induce organogenesis in the *Arabidopsis* inflorescence meristem [41]. Taken together, these results indicate that in stage 5, after exogenous GA_3_ treatment, crosstalk among GA, CTKs, and IAA likely led to the flower buds having a low CTK/IAA ratio and high GA level, which could have induced the formation of the lateral flower meristem in *P. callosum*.

**Table 2:** Effect of exogenous GA_3_ foliar spraying on the length and width of leaves in different developmental stages of *P. callosum*.

		Top-first leaf		Top-second leaf		Top-third leaf	
Stage	Treatment	Length (cm)	Width (cm)	Length (cm)	Width (cm)	Length (cm)	Width (cm)
4	Control	5.31 ± 0.26	0 ± 0	15.34 ± 0.73	3.55 ± 0.33	9.88 ± 0.23	2.48 ± 0.12
	GA_3_	6.23 ± 0.59	0 ± 0	18.41 ± 1.11	2.37 ± 0.35	14.65 ± 1.54	2.28 ± 0.44
5	Control	9.24 ± 0.63	0 ± 0	15.77 ± 0.59	3.57 ± 0.35	9.99 ± 0.36	2.64 ± 0.16
	GA_3_	12.63 ± 0.58	0 ± 0	20.87 ± 1.28^*^	2.58 ± 0.37	14.94 ± 1.27	2.37 ± 0.43
6	Control	11.54 ± 0.47	3.62 ± 0.18	15.32 ± 0.54	3.64 ± 0.21	10.80 ± 1.11	2.62 ± 0.19
	GA_3_	18.70 ± 0.47^*^	2.72 ± 0.11^*^	20.70 ± 1.01^*^	2.92 ± 0.09^*^	14.50 ± 1.54	2.67 ± 0.28

^*^Indicates a significant difference from the control (Student’s *t*-test, *P* < .05). Values are mean ± standard error.

### Effect of endogenous bioactive GAs on the expression of flowering- and bolting-related genes in the flower organ differentiation stage

Evidently, an exogenous GA_3_ application induces early flowering and increases the flower number of *P. callosum*. The pertinent question becomes which molecular mechanisms are responsible for this? To address this, the DEGs related to flowering were analyzed in depth (Fig. 7; Supplementary Data Table S5). The protein encoded by *SVP* is a key floral repressor, and GAs could induce the floral integrator *LFY* by repressing *SVP* via the GAF–TPR complex in the shoot apex, to promote flowering in *Arabidopsis* [42]. In *P. callosum*, *SVP* genes were expressed significantly less after the GA_3_ treatment in stage 4 and also in stage 5; however, we found no clear evidence for inflorescence initiation on the vegetative meristem being promoted in the GA_3_-treated group. Hence, the GA pathway was unlikely to be the main factor in controlling the floral transition of *Paphiopedilum*. By the end of stage 5, the dominant flower had differentiated into a complex floral organ, with its sepals, petals, and lip all visible; the RNA-Seq results from this stage showed that the A function gene *AP2*, the B function genes *AP3* and *PI*, and the *SEP* gene were significantly upregulated by the GA_3_ treatment. In *Arabidopsis,* it has been proven that GA promotes the expression of the floral homeotic genes *AP3*, *PI*, and *AG* by antagonizing the effects of DELLA proteins, thereby facilitating continued flower development [43]. In *P. callosum* a similar mechanism might exist.

Inflorescence stem elongation (i.e. bolting) is a critical event that occurs during plant reproductive development. In spinach and cabbage, rosette leaves keep growing under short-day conditions and bolting and flowering occur under long-day conditions, and exogenous GA could make plants bolt and flower under short-day conditions [44, 45]. Yet *Paphiopedilum* spp. are mainly distributed in tropical Asia, implying they are indifferent to day length and thus lack any strong photoperiodic responses [46]. The stem of *Paphiopedilum* is abbreviated, with the distichous leaf bases usually obscuring the stem. There is an apical meristem that continually differentiates leaves until its initiation of flower buds, and typically the elongation of the inflorescence stem is accompanied by the differentiation of floral organs. Our results showed that exogenous GA_3_ could function as a contributing factor in the control of inflorescence stem elongation, but it was apparently not essential for flower formation. In *Arabidopsis* flowering, the length and activity of its rib meristems were augmented, which enabled lengthening of the internodes forming underneath and between the floral buds [47]. Plants must coordinate rib cell renewal with daughter cell proliferation/differentiation and inflorescence development, so that the flowers emerge at the optimal time, height, and developmental stage [48]. GA can promote rib meristem-driven secondary axial elongation by hastening cell division and cell elongation processes [49].

In stage 5 of *P. callosum*, the high accumulation of bioactive GA coupled with the degradation of DELLA proteins induced by exogenous GA_3_ treatment would release the expression inhibition of TCP15 transcription factors. This upregulation of *TCP15* would have then stimulated several important cell-cycle genes and spurred cell division. Beyond this, exogenous GA_3_ promoted the expression of *XTH9*, leading to adjustment in cell wall flexibility and greater cell elongation of the inflorescence stem.

### Probable mechanism by which exogenous GA_3_ regulates flowering in *P. callosum*

**Figure 9:**
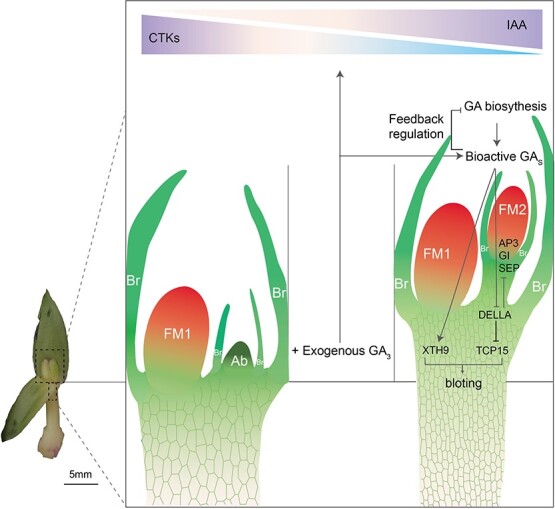
Proposed working model for the role of GA_3_ in promoting lateral flower formation and bolting in *P. callosum*. In stage 5, exogenous GA_3_ application stimulates the synthesis of endogenous GA_3_ and is accompanied by a decreasing level of CTKs and an increasing level of IAA. Endogenous levels of GA_3_ are fine-tuned via feedback control of GA-synthesis and GA-degradation genes. More bioactive GAs upregulate *PcXTH9* expression and thereby affect the binding activity of DELLA-TCP proteins with cell-cycle genes, thus promoting the bolting process. The low CTK/IAA ratio and high GA level, in combination with the greater expression of *PcAP3*, *PcPI*, and *PcSEP*, induce floral meristem formation, which contributes to lateral flowering in *P. callosum*. At the top is a color scale corresponding to multiples of increasing/decreasing levels of CTKs or IAA. Blue indicates a low level and purple indicates a high level. FM1, dominant flower; FM2, lateral flower; Ab, aborted bud; Br, bract.

Endogenous levels of GA are fine-tuned by a negative feedback regulatory mechanism that controls not only GA-synthesis genes, namely *PcGA2ox1B*, *PcGA2ox1D*, *PcGA3ox1*, and *PcGA3ox2*, but also the GA-degradation genes *PcGA2ox1* and *PcGA2ox8* (Fig. 9)*.* More bioactive GAs would be expected to promote the expression of *PcXTH9* and the degradation of *PcDELLAs*. Accordingly, upregulation of *PcXTH9* would foster cell elongation. The degradation of *PcDELLAs* would release the expression of *PcTCP15*, which would stimulate several important cell-cycle genes and promote cell division [20]. They may work together to promote bolting in *P. callosum* and advance its onset of flowering. Further, exogenous GA_3_ application resulted in some floral homeotic genes, such as *PcAP3*, *PcPI*, and *PcSEP*, being upregulated in flower buds (Fig. 9). This implies that *P. callosum* harbors a mechanism similar to that in *Arabidopsis*. That is, GA might also promote the expression of floral homeotic genes by antagonizing the effects of DELLA proteins, thereby allowing the continuation of flower development [43].

High levels of bioactive GAs were accompanied by a decreased CTK level and an increased IAA level in stage 5. Accordingly, endogenous hormones in flower buds featured a low CTK/IAA ratio and high GA level, which was more apt to induce the formation of the lateral floral meristem (Fig. 9).

## Materials and methods

### Plant materials, growth conditions, and GA_3_ treatment

The *P. callosum* plants were grown in a greenhouse in the South China Botanical Garden, Guangzhou, China. These plants were potted in a substrate of Zhijing stone suitable for orchids (Northridge Enterprise Co. Ltd, Taiwan, China) and received no more than 800 μmol m^−2^ s^−1^ of natural light (maintained by a sunshade net). The average temperature of the greenhouse was 10–32°C, and the relative humidity was 70–98%. A total of 100 uniformly grown *P. callosum* plants were used, each having one new tiller bud at the base, and they were randomly divided into two groups (i.e. *n* = 50 per group). Plants in the first group were treated with 100 mg/l of GA_3_ once every 2 weeks. The GA_3_ (Sigma-Aldrich, St Louis, MO, USA) was dissolved in 50% (v/v) ethanol/water. The second grouped was treated with the same amount of distilled water as the control group. This experiment was conducted over a 7-month period (from 15 July 2019 to 15 February 2020). Each treatment application was sprayed onto the whole plant with a low-pressure hand-wand sprayer.

### Phenotypic and histological observations

Microstructural changes of each bud were examined at 40, 80, 140, and 180 DABT. From the GA_3_ treatment group and non-GA_3_-treated (i.e. control) group, flower buds from four stages were fixed for 24 h in a formalin acetic acid–alcohol solution (70% ethyl alcohol:37% formaldehyde:glacial acetic acid = 18:1:1). The sections were observed under a microscope (Nikon, E200, Japan) with micrographs captured by an HQ Image C630 digital camera. Details about the samples’ preparation and paraffin sectioning are described in Fang *et al*. [50].

According to our phenotypic and histological observations, the process by which a new vegetative shoot becomes a flowering shoot of *P. callosum* can be divided into eight consecutive stages (refer to Results section for details). Leaves in stage 6 from the GA_3_ treatment group and control were sampled. Adaxial epidermal cells of the top, middle, and basal regions were peeled, and a 25-mm^2^ block of each leaf was dissected from the center of each adaxial epidermis, after which the cells were observed and photographed under a microscope (Nikon, E200, Japan). Cell counts and length measurements were made using ImageJ software (http://rsb.info.nih.gov/ij/, NIH, MD, USA).

### Measuring levels of endogenous plant hormones

Freshly harvested flower buds (each 100 mg) collected at 80 and 140 DABT from plants subjected to GA_3_ (likewise from the control group) were ground into powder and extracted with an acetonitrile solution. All standards for this analysis and its measurements were purchased from Sigma (USA). Samples were dried with nitrogen oxides and reconstituted, and then the lower organic phase was injected into a liquid chromatography–tandem mass spectrometry (LC–MS–MS) system equipped with a Poroshell 120 SB-C18 column (Agilent, USA) and a triple quadruple-tandem mass spectrometer (Quattro Premier XE; Waters) to detect the hormone levels. Three biological replications were performed for each test.

### RNA extraction and transcriptome analysis

Flower buds collected at 40, 80, and 140 DABT from plants receiving exogenous GA_3_ (likewise from the control group) were dissected and immediately frozen in liquid nitrogen. Three biological replicates per developmental stage were collected from each group. Total RNA of each sample was isolated using RNAiso Plus (TaKaRa, Dalian, China) according to the manufacturer’s protocol. To avoid genomic DNA contamination, RNA was treated with RNase-free DNaseI (TaKaRa, Dalian, China). RNA quality and quantity were respectively analyzed using an Agilent 2100 Bioanalyzer (Agilent Technologies, Inc., Santa Clara, CA, USA) and a NanoDrop ND2000 (NanoDrop Thermo Scientific, Wilmington, DE, USA), respectively. Finally, 18 normalized floral cDNA libraries of *P. callosum* were constructed and sequenced by a HiSeq X Ten/NovaSeq 6000 sequencer (Illumina, San Diego, CA, USA) at the Shanghai Majorbio Bio-pharm Biotechnology Co., Ltd (Shanghai, China).

### *De novo* assembly, functional annotation of unigenes, and differentially expressed gene analysis

Raw data were filtered to remove any adapter sequences, low-quality reads (quality value Q ≤20 at the 3′ end), reads containing N (modular bases), and sequences whose length was too short (<30 bp), by using the tools SeqPrep (https://github.com/jstjohn/SeqPrep) and Sickle (https://github.com/najoshi/sickle). The remaining high-quality data were used to generate unigenes on the Trinity software platform (http://trinityrnaseq.github.io) [51]. Their functional annotations and classification were performed using the alignment search tool BLASTX (http://blast.ncbi.nlmm.nih.gov/Blast.cgi), applied to six public databases, namely, NR, Swiss-Prot, GO, COG, KEGG, and Pfam. The expression level of each transcript was calculated according to the method of fragments per kilobase of exon per million mapped reads (FPKM). To identify DEGs between libraries, the DESeq2 tool was used (http://www.bioconductor.org/packages/release/bioc/html/DESeq2.html). Only those unigenes with a *P*-value ≤.05 and a fold change ≥1.5 were designated as differentially expressed. The obtained DEGs were subjected to GO enrichment and KEGG enrichment analyses.

### Verification of gene expression using qRT–PCR

The RNA-Seq samples were reversed-transcribed into cDNA by using the One-Step gDNA Removal and cDNA Synthesis SuperMix kit (TransGen, Beijing, China). The reverse-transcription cDNA was diluted to 100 ng/μl for use in the subsequent experiments. These reactions were completed on an ABI 7500 Real-Time PCR System (Applied Biosystems, CA, USA) in a 20-μl final volume that contained 1 μl of cDNA mixed with 10 μl of Green qPCR SuperMix (TransGen, Beijing, China), 0.4 μl of each primer (10 μM), and 8.2 μl of ddH_2_O. Six housekeeping genes (*60s*, *EF2*, *HSP70*, *VHA-D*, *ATP*, and *Acy1*) were selected as suitable reference genes in the three development stages and two treatment groups of flower buds. The mean threshold (CT) of the candidate genes ranged from 22.21 to 28.34 (Supplementary Data Table S6). Because it was the most stable gene among the six candidate reference genes we considered, *60s* (TRINITY_DN24966_c0_g2) was used in this study’s formal analysis. The gene-specific primers for qRT–PCR were designed by Primer 5.0 and are listed in Supplementary Data Table S7. Relative expression levels were calculated by the 2^-ΔΔCt^ method [52].

## Supplementary Material

suppl_data_uhac091

## Data Availability

The raw data are accessible at NCBI under the BioProject PRJNA744344.
