# Supplementary material for: Exogenous GA3 promotes flowering in Paphiopedilum callosum (Orchidaceae) through bolting and lateral flower development regulation
Source: Hortic Res. 2022 Apr 22;9:uhac091. doi: 10.1093/hr/uhac091 (PMC9249578; doi:10.1093/hr/uhac091)
Supplement: suppl_data_uhac091 [file suppl_data_uhac091.zip › 1Supplementary Information.docx]

**Supplementary Information**

**Figure S1. One-flowered *P. callosum* with an aborted bud in the apical bract**

Ab: an aborted bud


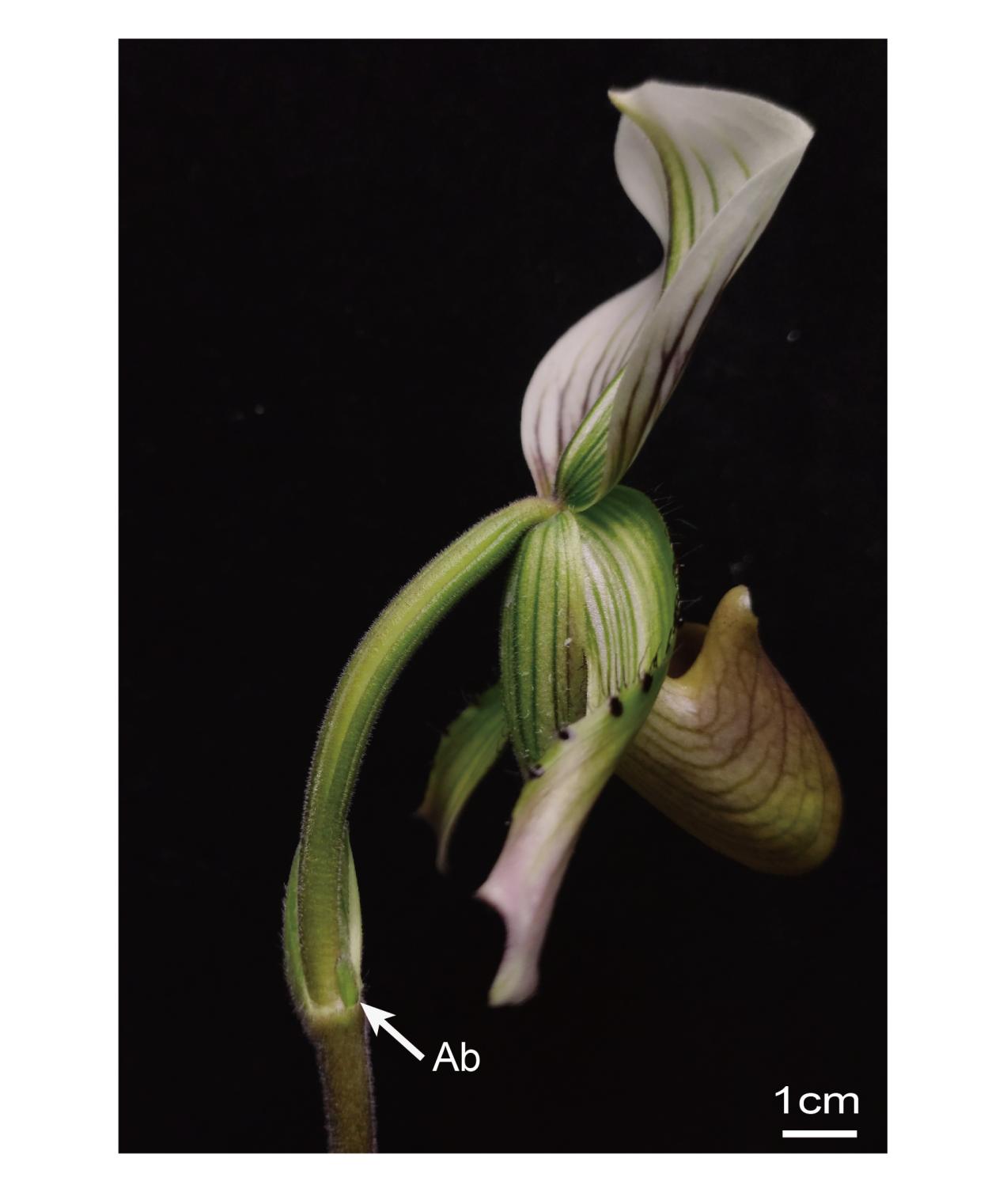


**Figure S2. Changes in the levels of endogenous hormones zeatin, IP, JA and SA from 80 DABT to 140 DABT between the GA_3_ treated *P. callosum* and control**

**
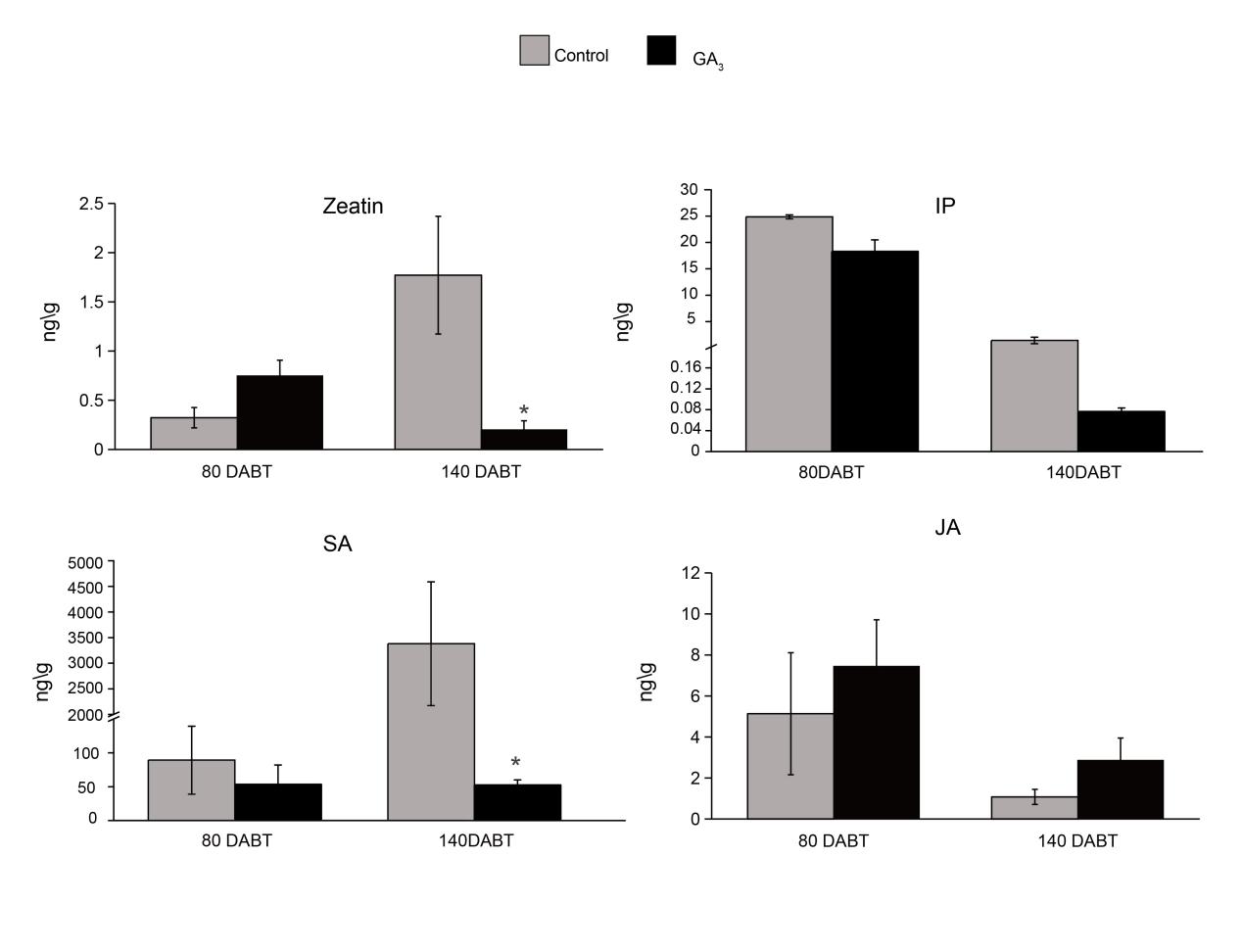
**

**Figure S3. (a) Principal component** **analysis (PCA) performed on the 18 samples. (b) NR annotated species distribution map.** *Dendrobium catenatum* shows the highest similarity.

**
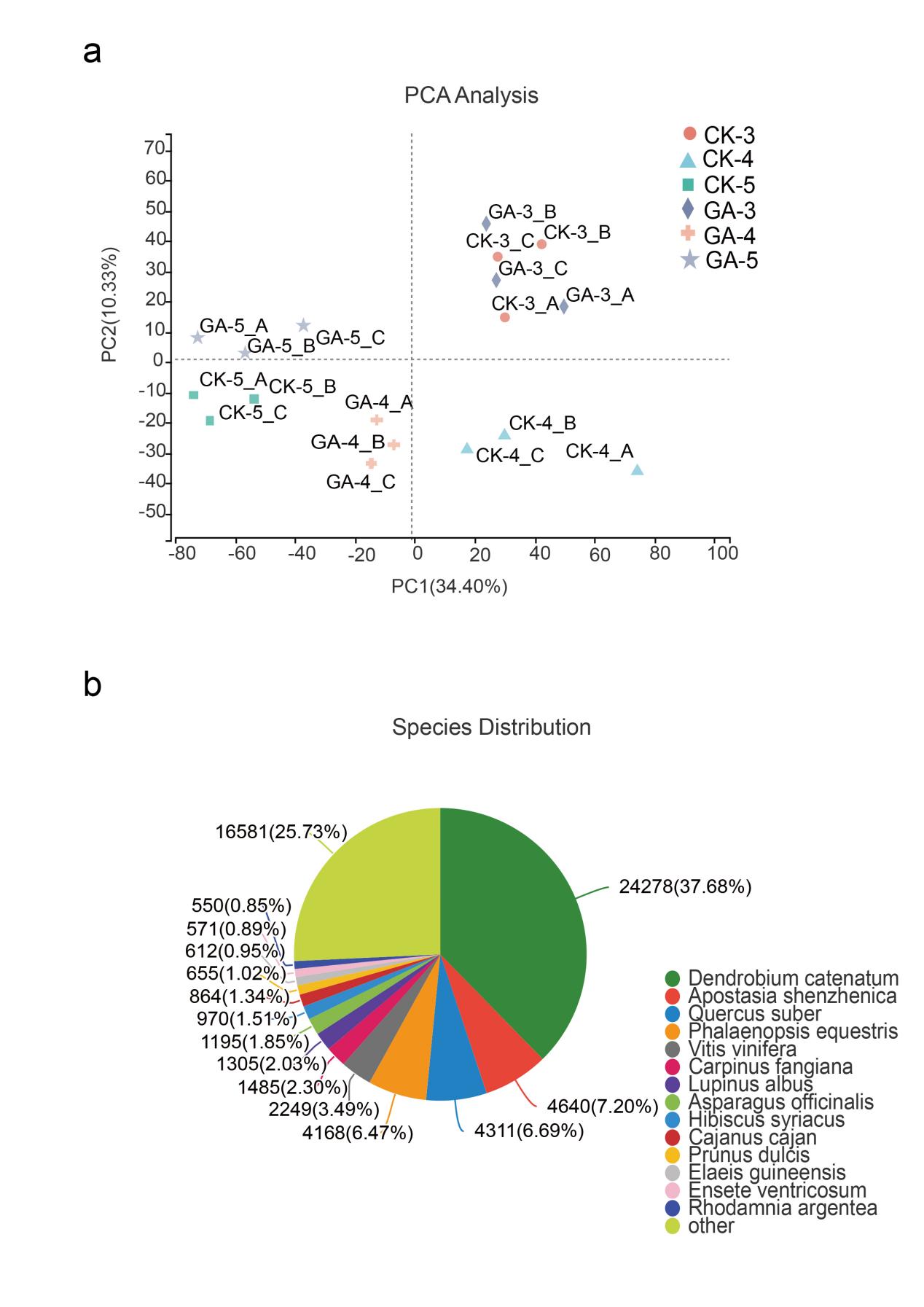
**

**Figure S4. (a)** **Multi-flowered type: flowering *P. dianthum* and paraffin section of its inflorescence. (b) One-flowered type: flowering *P. callosum* and paraffin section of its inflorescence.**

**
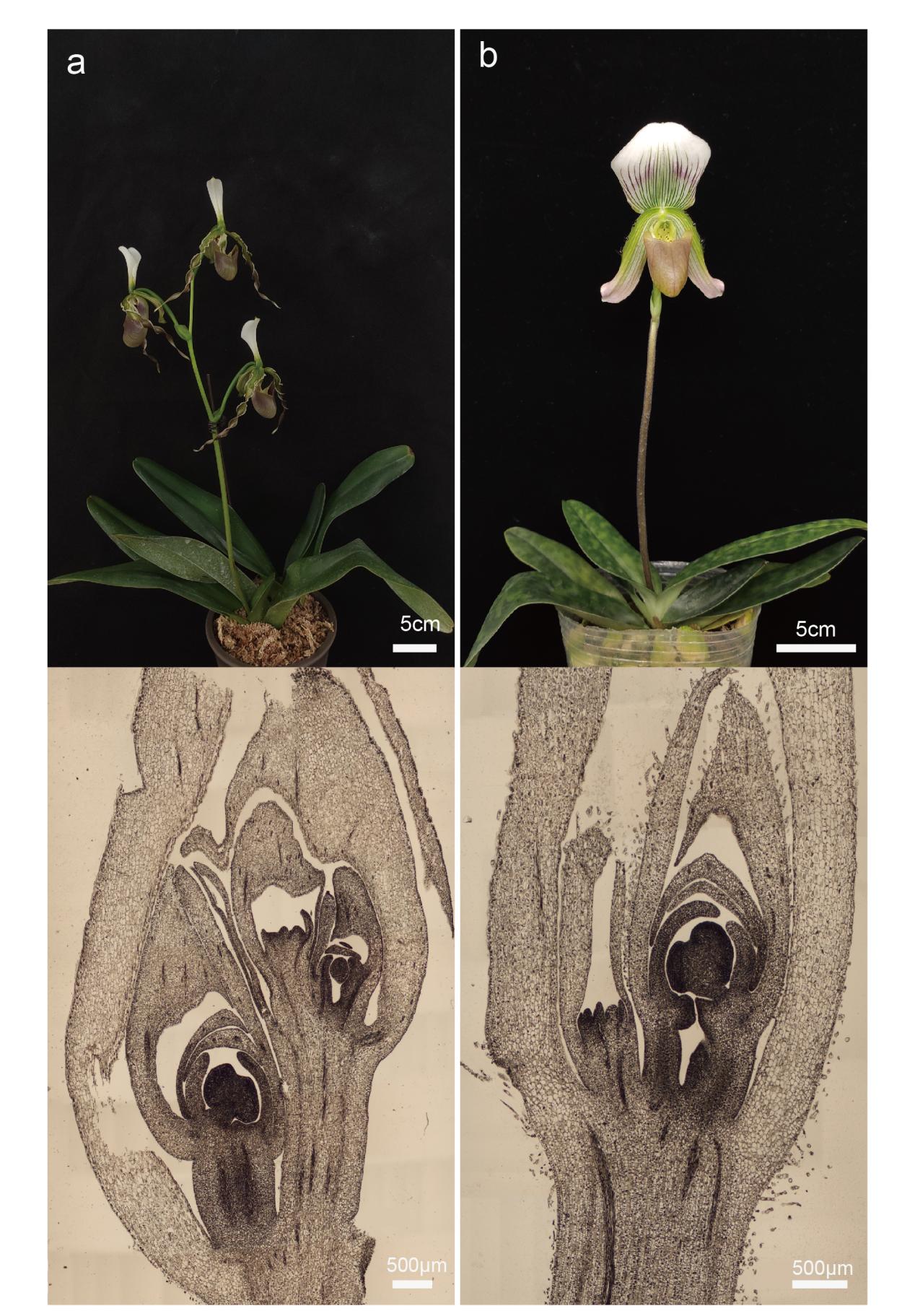
**

**Table S 1 A summary of the sequencing data**

| Sample | Raw reads | Clean reads | Mapped reads | Mapped ratio | Q20(%) | Q30(%) | GC content(%) |
| --- | --- | --- | --- | --- | --- | --- | --- |
| CK-3_A | 44490928 | 44019216 | 37907678 | 86.12% | 97.52 | 92.45 | 49.76 |
| CK-3_B | 57650956 | 56820880 | 47882354 | 84.27% | 98.13 | 94.68 | 52.37 |
| CK-3_C | 48477768 | 47608998 | 40901944 | 85.91% | 98.01 | 94.4 | 51.29 |
| CK-4_A | 43201454 | 43047800 | 37286470 | 86.62% | 98.28 | 94.82 | 49.36 |
| CK-4_B | 48534704 | 48361814 | 41460262 | 85.73% | 97.86 | 93.53 | 47.85 |
| CK-4_C | 44608830 | 44437456 | 38226300 | 86.02% | 97.8 | 93.52 | 47.41 |
| CK-5_A | 48925860 | 48614922 | 40071726 | 82.43% | 98.86 | 96.31 | 46.66 |
| CK-5_B | 49504710 | 49178332 | 41232766 | 83.84% | 98.86 | 96.31 | 46.99 |
| CK-5_C | 50410208 | 50078462 | 41473710 | 82.82% | 98.9 | 96.45 | 46.9 |
| GA-3_A | 43078550 | 42605674 | 36990292 | 86.82% | 97.85 | 93.39 | 51.43 |
| GA-3_B | 52978790 | 52027114 | 44037966 | 84.64% | 98.01 | 94.42 | 51.33 |
| GA-3_C | 46941744 | 46097148 | 39786892 | 86.31% | 98.07 | 94.54 | 51 |
| GA-4_A | 46541472 | 46388632 | 40072058 | 86.38% | 97.71 | 92.82 | 47.08 |
| GA-4_B | 37669104 | 37554384 | 32386064 | 86.24% | 97.4 | 91.93 | 47.09 |
| GA-4_C | 47793658 | 47647770 | 41218318 | 86.51% | 97.83 | 93.07 | 47.18 |
| GA-5_A | 49543912 | 49264664 | 40874646 | 82.97% | 98.94 | 96.57 | 46.91 |
| GA-5_B | 52478566 | 52173684 | 43810500 | 83.97% | 98.94 | 96.59 | 47.38 |
| GA-5_C | 52986816 | 52662114 | 44237334 | 84.00% | 98.92 | 96.48 | 46.75 |

**Table S 2 The detailed results of de novo assembly**

| Type | Unigene | Transcript |
| --- | --- | --- |
| Total number | 157463 | 233325 |
| Total base | 127782719 | 225857708 |
| Largest length (bp) | 18204 | 18204 |
| Smallest length (bp) | 201 | 201 |
| Average length (bp) | 811.51 | 968 |
| N50 length (bp) | 1282 | 1707 |
| E90N50 length (bp) | 3147 | 2433 |
| Fragment mapped percent(%) | 65.928 | 85.325 |
| GC percent (%) | 41.42 | 41.61 |

**Table S 3 Annotated unigenes statistics of different databases**

| Annotated databases | Number of annotated unigene | Percentage (%) |
| --- | --- | --- |
| NR | 64434 | 40.92 |
| Swiss-Prot | 39469 | 25.07 |
| GO | 53444 | 33.94 |
| COG | 45973 | 29.2 |
| KEGG | 23864 | 15.16 |
| Pfam | 39651 | 25.18 |
| Total_anno | 69997 | 44.45 |

**Table S 4 Annotated genes related to major hormone metabolism and signaling**

| Stage | Plant hormone signal transduction | | Gene Name | Unigene ID | Description | Log2FC(GA2/CK2) | p_value | Regulate | CK_A | CK_B | CK_C | GA_A | GA_B | GA_C |
| --- | --- | --- | --- | --- | --- | --- | --- | --- | --- | --- | --- | --- | --- | --- |
| Stage 3 | Gibberellin | Synthesis | GA20ox1B | TRINITY_DN11321_c0_g1 | Gibberellin 20 oxidase 1-B [Dendrobium catenatum] | -1.787237086 | 0.004785822 | down | 4.03 | 18.5 | 17.66 | 1.56 | 8.24 | 3.79 |
|  |  |  | GA2OX1 | TRINITY_DN9023_c0_g2 | Gibberellin 2-beta-dioxygenase 2 [Dendrobium catenatum] | 1.031905104 | 0.031235497 | up | 2.67 | 1.15 | 2.82 | 4.27 | 3.28 | 4.85 |
|  |  |  | GA2OX1 | TRINITY_DN3903_c0_g2 | Gibberellin 2-beta-dioxygenase [Dendrobium catenatum] | 0.921197565 | 0.03238838 | up | 8.59 | 13.18 | 20.45 | 21.77 | 32.2 | 31 |
|  |  | Signalling | GID1C | TRINITY_DN6855_c0_g1 | gibberellin receptor GID1C-like isoform X1 [Phalaenopsis equestris] | -0.87096584 | 0.045460536 | down | 11.23 | 19.04 | 18.88 | 9.85 | 11.24 | 7.58 |
|  |  |  | DELLA1 | TRINITY_DN117929_c0_g1 | DELLA protein GAI1 [Dendrobium catenatum] | -1.053325518 | 0.018956855 | down | 29.1 | 43.02 | 25.39 | 11.5 | 24.24 | 15.9 |
|  | Auxin | Synthesis | YUCCA4 | TRINITY_DN9731_c0_g1 | putative indole-3-pyruvate monooxygenase YUCCA4 [Dendrobium catenatum] | -2.241860079 | 0.003462281 | down | 0.75 | 1.07 | 2.42 | 0.36 | 0.41 | 0.13 |
|  |  | Signalling | AUX22D | TRINITY_DN37926_c0_g1 | auxin-induced protein 22D [Dendrobium catenatum] | 0.95580363 | 0.014941325 | up | 18.23 | 7.43 | 17.61 | 23.41 | 33.56 | 23.47 |
|  |  |  | SAUR40 | TRINITY_DN3733_c0_g1 | Indole-3-acetic acid-induced protein ARG7 [Apostasia shenzhenica] | 1.027126592 | 0.045085308 | up | 8.91 | 2.93 | 14.49 | 23.56 | 12.32 | 13.88 |
|  |  |  | SAUR50 | TRINITY_DN11229_c0_g1 | auxin-responsive protein SAUR50-like [Phalaenopsis equestris] | 2.53055533 | 4.90376E-07 | up | 29.82 | 8.24 | 46.98 | 229.57 | 113.92 | 109.88 |
|  |  |  | SAUR71 | TRINITY_DN7508_c0_g1 | Auxin-induced protein X10A [Apostasia shenzhenica] | -2.103232558 | 0.022457916 | down | 2.62 | 9.81 | 6.87 | 0.4 | 4.6 | 0.52 |
|  |  |  | SAUR72 | TRINITY_DN7738_c0_g1 | auxin-induced protein X10A-like [Phalaenopsis equestris] | 1.216833496 | 0.032602962 | up | 12.83 | 2.4 | 7.36 | 26.62 | 11.68 | 8.26 |
|  | Cytokinine | Signalling | RR10 | TRINITY_DN3265_c0_g1 | PREDICTED: two-component response regulator ORR10-like isoform X1 [Nicotiana tabacum] | -0.895935126 | 0.023211199 | down | 13.81 | 13.15 | 11.62 | 6.61 | 7.78 | 7.24 |
|  | Abscisic acid | Degradation | CYP707A7 | TRINITY_DN1092_c0_g1 | abscisic acid 8'-hydroxylase 3 [Dendrobium catenatum] | -1.36295499 | 0.006637886 | down | 5.35 | 9.16 | 9.38 | 3.09 | 2.01 | 4.6 |
| Stage 4 |  | Synthesis | KAO1 | TRINITY_DN25259_c0_g1 | ent-kaurenoic acid oxidase 2 [Dendrobium catenatum] | 0.60703336 | 0.012473197 | up | 27.86 | 32.86 | 36.3 | 60.63 | 59.73 | 48.4 |
|  |  |  | GA20ox1D | TRINITY_DN23906_c1_g2 | gibberellin 20 oxidase 1-D [Dendrobium catenatum] | -1.413396431 | 0.000197094 | down | 235.09 | 128.89 | 106.01 | 83.01 | 79.2 | 66.56 |
|  |  |  | GA20ox1B | TRINITY_DN11321_c0_g1 | Gibberellin 20 oxidase 1-B [Dendrobium catenatum] | -2.119175036 | 0.000247182 | down | 2.76 | 2.16 | 2.57 | 0.58 | 0.67 | 0.75 |
|  |  |  | GA3OX2 | TRINITY_DN89195_c1_g1 | gibberellin 3-oxidase [Dendrobium officinale] | -1.556789321 | 0.01134996 | down | 2.65 | 3.17 | 2.53 | 0.75 | 0.66 | 2.03 |
|  |  |  | GA3OX1 | TRINITY_DN16323_c0_g1 | gibberellin 3-beta-dioxygenase 1-like [Phalaenopsis equestris] | -0.96716081 | 0.013921992 | down | 6.78 | 6.05 | 6.67 | 3.73 | 4.61 | 4.13 |
|  |  | Degradation | GA2OX1 | TRINITY_DN9023_c0_g2 | Gibberellin 2-beta-dioxygenase 2 [Dendrobium catenatum] | 0.822237117 | 0.031312425 | up | 2.69 | 3.18 | 3.49 | 7.15 | 6.25 | 7.25 |
|  |  | Signalling | GAI | TRINITY_DN71_c0_g1 | DELLA protein SLN1 [Dendrobium catenatum] | -0.662568354 | 0.045260443 | down | 19.07 | 17.71 | 19.53 | 19.83 | 17.12 | 13.77 |
|  |  |  | GID2 | TRINITY_DN11275_c0_g1 | F-box protein GID2 [Phalaenopsis equestris] | -1.209141246 | 0.000144543 | down | 52.78 | 44 | 42.53 | 23.33 | 26.03 | 27.38 |
|  | Auxin | Synthesis | YUCCA8 | TRINITY_DN9731_c0_g1 | putative indole-3-pyruvate monooxygenase YUCCA4 [Dendrobium catenatum] | -3.290686651 | 2.65564E-06 | down | 1.24 | 3.34 | 2.06 | 0.11 | 0.5 | 0.2 |
|  |  |  | YUCCA2 | TRINITY_DN35558_c0_g2 | LOW QUALITY PROTEIN: indole-3-pyruvate monooxygenase YUCCA2, partial [Dendrobium catenatum] | -1.400265172 | 0.021869774 | down | 0.94 | 1.28 | 2.69 | 0.41 | 0.69 | 1.02 |
|  |  | Transport | PIN2 | TRINITY_DN2184_c1_g1 | putative auxin efflux carrier component 2 [Apostasia shenzhenica] | 0.646117268 | 0.01881493 | up | 1.88 | 4.01 | 5.71 | 8.55 | 7.7 | 6.56 |
|  |  | Signalling | AUX22D | TRINITY_DN37926_c0_g1 | auxin-induced protein 22D [Dendrobium catenatum] | 1.07663154 | 0.003201627 | up | 17.69 | 12.49 | 14.12 | 32.92 | 38.72 | 47.39 |
|  |  |  | ARF9 | TRINITY_DN1973_c0_g2 | auxin response factor 9-like isoform X1, partial [Cymbidium sinense] | -0.605921263 | 0.024966105 | down | 14.78 | 17.52 | 17.34 | 13.1 | 13.36 | 12.99 |
|  |  |  | ARF18 | TRINITY_DN27648_c0_g2 | Auxin response factor 11 [Apostasia shenzhenica] | 1.945207162 | 0.041229729 | up | 0 | 0.27 | 0.64 | 1.08 | 2.29 | 0.4 |
|  |  |  | SAUR32 | TRINITY_DN7738_c0_g1 | auxin-induced protein X10A-like [Phalaenopsis equestris] | -1.264327543 | 0.012227805 | down | 16.08 | 9.72 | 9 | 5.58 | 4.4 | 9.16 |
|  |  |  | SAUR32 | TRINITY_DN10793_c0_g1 | Auxin-induced protein X10A [Dendrobium catenatum] | -1.773743898 | 0.004424548 | down | 39.8 | 17.27 | 7.43 | 9.6 | 11.15 | 7.37 |
|  |  |  | SAUR32 | TRINITY_DN6045_c0_g1 | auxin-responsive protein SAUR32 [Dendrobium catenatum] | -1.814670578 | 0.000259002 | down | 15.21 | 9.88 | 11.86 | 6.78 | 3.91 | 3.02 |
|  |  |  | SAUR50 | TRINITY_DN11229_c0_g1 | auxin-responsive protein SAUR50-like [Phalaenopsis equestris] | 1.64791748 | 4.2176E-10 | up | 24.15 | 46.23 | 48.23 | 145.05 | 139.5 | 158.99 |
|  |  |  | SAUR71 | TRINITY_DN133900_c0_g1 | Auxin-induced protein X15 [Apostasia shenzhenica] | -0.941674458 | 0.044426969 | down | 11.65 | 7.94 | 6.89 | 6.88 | 6.66 | 4.62 |
|  |  |  | GH3.8 | TRINITY_DN13041_c0_g1 | probable indole-3-acetic acid-amido synthetase GH3.8 [Dendrobium catenatum] | -1.213358201 | 0.033449858 | down | 4.14 | 2.29 | 1.55 | 2.15 | 1.74 | 0.83 |
|  |  |  | IAA20 | TRINITY_DN4971_c0_g1 | auxin-responsive protein IAA20 isoform X2 [Elaeis guineensis] | 1.229288866 | 2.57356E-05 | up | 27.4 | 23.37 | 21.48 | 69.77 | 56.53 | 61.8 |
|  | Cytokinine | Degradation | CKX3 | TRINITY_DN54775_c0_g4 | cytokinin dehydrogenase 3-like [Phalaenopsis equestris] | -1.072019934 | 0.000623888 | down | 4.31 | 6.73 | 7.91 | 3.42 | 3.49 | 3.7 |
|  |  |  | CKX5 | TRINITY_DN11731_c0_g1 | cytokinin dehydrogenase 5 [Dendrobium catenatum] | -0.931113962 | 0.005828867 | down | 5.49 | 5.52 | 7.09 | 3.56 | 4.14 | 3.69 |
|  |  |  | CKX11 | TRINITY_DN3125_c0_g1 | cytokinin dehydrogenase 11 isoform X1 [Dendrobium catenatum] | -1.002625096 | 0.000433378 | down | 18.01 | 17.52 | 19.3 | 11.07 | 9.75 | 11.8 |
|  |  | Signalling | AHP1 | TRINITY_DN3989_c0_g1 | histidine-containing phosphotransfer protein 1 [Phalaenopsis equestris] | -0.640702572 | 0.03578283 | down | 67.98 | 67.92 | 57.64 | 44.73 | 52.35 | 59.47 |
|  |  |  | AHP2 | TRINITY_DN11026_c0_g2 | histidine-containing phosphotransfer protein 2-like [Phalaenopsis equestris] | -0.718741934 | 0.014700464 | down | 81.77 | 87.45 | 82.74 | 54.79 | 57 | 79.42 |
|  |  |  | RR2 | TRINITY_DN5392_c0_g1 | two-component response regulator ORR1-like isoform X1 [Phalaenopsis equestris] | -0.745181251 | 0.028814424 | down | 26.1 | 26.42 | 21.52 | 15.77 | 15.51 | 24.13 |
|  |  |  | RR9 | TRINITY_DN713_c0_g1 | two-component response regulator ORR9 [Dendrobium catenatum] | -0.688333087 | 0.016704067 | down | 18.64 | 32.21 | 36.18 | 16.2 | 19.03 | 24.28 |
|  |  |  | RR10A | TRINITY_DN26880_c0_g1 | two-component response regulator RR10 [Oncidium sp. BY-2020] | -2.418405777 | 2.15959E-06 | down | 4.75 | 4.63 | 8.34 | 1.77 | 1.18 | 1.04 |
|  |  |  | RR10A | TRINITY_DN3265_c0_g1 | PREDICTED: two-component response regulator ORR10-like isoform X1 [Nicotiana tabacum] | -1.366930776 | 2.8318E-05 | down | 10.31 | 19.57 | 23.31 | 8.21 | 8.44 | 7.85 |
|  | Abscisic acid | Synthesis | NCED | TRINITY_DN1548_c1_g1 | 9-cis-epoxycarotenoid dioxygenase, chloroplastic [Dendrobium catenatum] | -2.268857615 | 7.54937E-05 | down | 0.66 | 2.2 | 3.52 | 0.64 | 0.54 | 0.3 |
|  |  |  | NCED | TRINITY_DN1548_c0_g2 | 9-cis-epoxycarotenoid dioxygenase, chloroplastic [Dendrobium catenatum] | -1.305756715 | 0.00291423 | down | 3.21 | 5.17 | 7.01 | 3.32 | 3.01 | 1 |
|  |  |  | NCED | TRINITY_DN1548_c0_g1 | 9-cis-epoxycarotenoid dioxygenase, chloroplastic [Dendrobium catenatum] | -1.142097897 | 0.041628914 | down | 0.92 | 1.18 | 1.07 | 0.67 | 0.42 | 0.64 |
|  |  |  | CYP707A1 | TRINITY_DN129_c0_g1 | abscisic acid 8'-hydroxylase CYP707A2-like [Dendrobium catenatum] | 1.733305614 | 0.001390039 | up | 3.44 | 3.34 | 3.07 | 23.3 | 17.15 | 2.2 |
|  |  | Signalling | PP2CA | TRINITY_DN11018_c0_g2 | probable protein phosphatase 2C 68 [Dendrobium catenatum] | -1.599989837 | 3.08075E-09 | down | 11.23 | 16.28 | 16.79 | 5.77 | 6 | 5.46 |
|  |  |  | PP2C51 | TRINITY_DN2622_c0_g1 | probable protein phosphatase 2C 24 [Phalaenopsis equestris] | -0.968948152 | 0.001435846 | down | 53.71 | 46.29 | 40.38 | 31.39 | 29.64 | 29.79 |
|  |  |  | PP2CA | TRINITY_DN6611_c1_g3 | protein phosphatase 2C 37-like [Phalaenopsis equestris] | -0.899843982 | 0.00270332 | down | 15.59 | 14.58 | 15.76 | 12.12 | 10.17 | 8.24 |
|  |  |  | PP2CA | TRINITY_DN8590_c1_g3 | putative protein phosphatase 2C 8 [Apostasia shenzhenica] | -0.613060238 | 0.015868733 | down | 26.38 | 46.53 | 50.67 | 32.87 | 31.09 | 32.16 |
| Stage 5 | Gibberellin | Synthesis | GA20ox1B | TRINITY_DN11321_c0_g1 | Gibberellin 20 oxidase 1-B [Dendrobium catenatum] | -3.274727649 | 6.2349E-15 | down | 9.75 | 4.32 | 4.59 | 0.74 | 0.63 | 0.59 |
|  |  |  | GA20ox1D | TRINITY_DN21950_c1_g1 | gibberellin 20 oxidase 1-D-like [Dendrobium catenatum] | -4.575068045 | 2.86617E-06 | down | 1.28 | 0.86 | 1.05 | 0.13 | 0 | 0 |
|  |  |  | GA3OX1 | TRINITY_DN89195_c1_g1 | gibberellin 3-oxidase [Dendrobium officinale] | -2.666218294 | 0.000125903 | down | 1.62 | 1.41 | 1.15 | 0.14 | 0.34 | 0.19 |
|  |  |  | GA3OX2 | TRINITY_DN16323_c0_g1 | gibberellin 3-beta-dioxygenase 1-like [Phalaenopsis equestris] | -1.917453366 | 0.00399177 | down | 0.64 | 6.92 | 2.43 | 0.75 | 1.3 | 0.72 |
|  |  | Inactivation | GA2OX1 | TRINITY_DN3903_c0_g2 | Gibberellin 2-beta-dioxygenase [Dendrobium catenatum] | 1.339257838 | 1.49285E-07 | up | 4.87 | 4.96 | 4.86 | 12.78 | 14.67 | 11.03 |
|  |  |  | GA2OX1 | TRINITY_DN11448_c0_g1 | Gibberellin 2-beta-dioxygenase 1 [Dendrobium catenatum] | 1.341031774 | 0.016053243 | up | 2.48 | 3.62 | 1.24 | 5.34 | 7.23 | 4.45 |
|  |  |  | GA2OX8 | TRINITY_DN5120_c0_g1 | gibberellin 2-beta-dioxygenase 8-like [Dendrobium catenatum] | 4.506694607 | 4.35348E-23 | up | 0.22 | 1.03 | 0.28 | 9.35 | 14.5 | 10.19 |
|  |  | Signalling | GID1C | TRINITY_DN7924_c0_g1 | Gibberellin receptor GID1C [Apostasia shenzhenica] | -0.755739942 | 0.000585582 | down | 72.36 | 90.71 | 93.37 | 52.21 | 57.05 | 49.29 |
|  |  |  | GID1C | TRINITY_DN781_c0_g1 | gibberellin receptor GID1C-like isoform X1 [Phalaenopsis equestris] | -1.16921035 | 1.99314E-06 | down | 11.38 | 13.82 | 10.8 | 5.61 | 4.65 | 6.66 |
|  |  |  | SLR1 | TRINITY_DN1701_c0_g1 | DELLA protein SLR1-like [Phalaenopsis equestris] | 1.188686307 | 3.90469E-08 | up | 71.92 | 80.46 | 66.73 | 170.01 | 196.32 | 141.5 |
|  |  |  | GAI1 | TRINITY_DN55894_c0_g2 | DELLA protein GAI [Apostasia shenzhenica] | 0.976869129 | 0.000763307 | up | 2.73 | 3.58 | 2.54 | 7.24 | 5.77 | 5.2 |
|  |  |  | DWRF8 | TRINITY_DN7922_c0_g1 | DELLA protein SLN1-like isoform X4 [Cymbidium goeringii] | 0.667350821 | 0.000851134 | down | 67.02 | 74.73 | 64.36 | 45.79 | 43.71 | 35.9 |
|  |  |  | DELLA1 | TRINITY_DN117929_c0_g1 | DELLA protein GAI1 [Dendrobium catenatum] | 1.425946426 | 0.006827178 | down | 4.7 | 2.04 | 1.94 | 0.74 | 1.91 | 0.4 |
|  |  |  | GID2 | TRINITY_DN11275_c0_g1 | F-box protein GID2 [Phalaenopsis equestris] | -0.838662764 | 0.001064814 | down | 21.61 | 31.69 | 25.85 | 14.52 | 14.85 | 16.64 |
|  | Auxin | Degradation | DAO | TRINITY_DN1476_c0_g1 | 2-oxoglutarate-dependent dioxygenase DAO-like [Asparagus officinalis] | -2.959277019 | 2.78688E-23 | down | 143.27 | 110.74 | 111.63 | 11.18 | 11.96 | 25.35 |
|  |  |  | DAO | TRINITY_DN30748_c0_g1 | 2-oxoglutarate-dependent dioxygenase DAO-like [Phalaenopsis equestris] | -3.821286164 | 1.61064E-09 | down | 2.1 | 2.6 | 3.14 | 0.25 | 0.1 | 0.24 |
|  |  | Transport | PIN3A | TRINITY_DN2262_c0_g1 | auxin efflux carrier component 4 [Dendrobium catenatum] | 0.717107721 | 0.025353244 | up | 13.87 | 12 | 7.02 | 13.74 | 27.22 | 14.57 |
|  |  |  | PIN1C | TRINITY_DN1322_c0_g1 | LOW QUALITY PROTEIN: probable auxin efflux carrier component 1b [Phalaenopsis equestris] | 1.420787329 | 1.20651E-06 | up | 21.01 | 15.81 | 9.34 | 43.56 | 52.36 | 30.38 |
|  |  |  | LAX5 | TRINITY_DN3289_c1_g1 | auxin transporter-like protein 2 [Phalaenopsis equestris] | 0.888595361 | 0.006676221 | up | 29.61 | 58.86 | 18.99 | 71.61 | 64.13 | 71.22 |
|  |  | Signalling | IAA4 | TRINITY_DN6756_c0_g1 | auxin-induced protein 22D [Dendrobium catenatum] | 1.610180997 | 6.02726E-05 | up | 6.7 | 6.19 | 11.92 | 29.19 | 37.28 |  |
|  |  |  | AUX22D | TRINITY_DN37926_c0_g1 | auxin-induced protein 22D [Dendrobium catenatum] | 0.976293689 | 0.011329863 | up | 12 | 4.23 | 12.15 | 14.67 | 23.58 | 19.61 |
|  |  |  | ARF11 | TRINITY_DN1973_c1_g1 | auxin response factor 9-like [Phalaenopsis equestris] | -0.725348214 | 0.018536662 | down | 42.03 | 44.4 | 71.11 | 36.19 | 19.61 | 45.2 |
|  |  |  | ARF15 | TRINITY_DN2496_c0_g1 | auxin response factor 3-like isoform X1 [Dendrobium catenatum] | -0.761243061 | 0.000165065 | down | 56.09 | 41.67 | 43.73 | 27.94 | 26.81 | 30.54 |
|  |  |  | SAUR32 | TRINITY_DN10793_c0_g1 | Auxin-induced protein X10A [Dendrobium catenatum] | -2.236409666 | 6.68612E-05 | down | 12.31 | 6.51 | 8.19 | 1.64 | 1.37 | 2.78 |
|  |  |  | SAUR32 | TRINITY_DN8060_c0_g1 | Auxin-induced protein X15 [Apostasia shenzhenica] | -2.40951291 | 6.64696E-07 | down | 3.58 | 4.35 | 5.14 | 1.38 | 0.27 | 1.05 |
|  |  |  | SAUR50 | TRINITY_DN11229_c0_g1 | auxin-responsive protein SAUR50-like [Phalaenopsis equestris] | 2.396375708 | 2.66179E-19 | up | 22.85 | 21.99 | 14.97 | 81.94 | 105.07 | 134.3 |
|  |  |  | SAUR71 | TRINITY_DN133900_c0_g1 | Auxin-induced protein X15 [Apostasia shenzhenica] | 1.055319399 | 0.00817425 | up | 2.43 | 3.65 | 6.07 | 8.07 | 8.68 | 9.63 |
|  |  |  | IAA2 | TRINITY_DN9630_c0_g1 | auxin-responsive protein IAA2 [Dendrobium catenatum] | -0.639244864 | 0.009791848 | down | 20.69 | 31.71 | 26.52 | 19.87 | 14.06 | 19.81 |
|  |  |  | IAA6 | TRINITY_DN174_c0_g1 | auxin-responsive protein IAA26 [Dendrobium catenatum] | -1.189554684 | 1.39127E-05 | down | 7.7 | 9 | 6.52 | 3.67 | 2.81 | 4.09 |
|  | Cytokinine | Synthesis | IPT1 | TRINITY_DN20131_c0_g1 | adenylate isopentenyltransferase-like [Dendrobium catenatum] | -2.227472199 | 7.49663E-06 | down | 1.85 | 3.19 | 2.75 | 1.08 | 0.43 | 0.26 |
|  |  |  | CYP735A1 | TRINITY_DN20354_c0_g1 | cytokinin hydroxylase-like [Dendrobium catenatum] | -4.472327154 | 2.71952E-18 | down | 10.23 | 2.21 | 8.16 | 0.44 | 0.36 | 0.24 |
|  |  | Signalling | AHP1 | TRINITY_DN3989_c0_g1 | histidine-containing phosphotransfer protein 1 [Phalaenopsis equestris] | -0.870203356 | 5.45728E-05 | down | 48.6 | 46.44 | 48.08 | 25.62 | 25.27 | 30.27 |
|  |  |  | RR9 | TRINITY_DN2495_c0_g1 | two-component response regulator ORR9 [Dendrobium catenatum] | -1.948170258 | 2.9272E-15 | down | 24.85 | 23.4 | 25.28 | 5.1 | 6.76 | 8.37 |
|  |  |  | RR9 | TRINITY_DN26880_c0_g1 | two-component response regulator RR10 [Oncidium sp. BY-2020] | -2.434966465 | 1.01821E-09 | down | 6.6 | 7.82 | 7.25 | 0.98 | 1.88 | 1.25 |
|  |  |  | RR10 | TRINITY_DN713_c0_g1 | two-component response regulator ORR9 [Dendrobium catenatum] | -1.898190177 | 2.15735E-13 | down | 76.88 | 42.02 | 59.8 | 14.26 | 15.47 | 22.85 |
|  |  |  | RR10 | TRINITY_DN3265_c0_g1 | PREDICTED: two-component response regulator ORR10-like isoform X1 [Nicotiana tabacum] | -2.078815095 | 1.15863E-11 | down | 49.05 | 58.41 | 62.18 | 8.47 | 17.77 | 14.83 |
|  | Abscisic acid | Degradation | CYP707A7 | TRINITY_DN39421_c0_g3 | abscisic acid 8'-hydroxylase 3 [Dendrobium catenatum] | -2.90391039 | 5.20449E-13 | down | 4.94 | 3.47 | 7.09 | 0.47 | 0.92 | 0.75 |
|  |  |  | CYP707A5 | TRINITY_DN129_c0_g1 | abscisic acid 8'-hydroxylase CYP707A2-like [Dendrobium catenatum] | 1.995116461 | 0.00705094 | up | 5.38 | 12.89 | 5.67 | 65.15 | 20.23 | 14.8 |
|  |  | Signalling | ABF2 | TRINITY_DN16513_c0_g1 | putative ABSCISIC ACID-INSENSITIVE 5-like protein 5 isoform X1, partial [Cymbidium ensifolium] | -1.029205035 | 0.000432656 | down | 32.73 | 48.32 | 61.84 | 33.03 | 18.35 | 21.53 |

**Table S 5 Annotated genes related to flowering and bloting**

| Stage | Locus | CK | GA_3_ | Regulate | Log2FC(GA/CK) | p_value | Description | keyword |
| --- | --- | --- | --- | --- | --- | --- | --- | --- |
| stageⅤ | TRINITY_DN55824_c0_g1 | 4.32 | 1.793333333 | down | -1.628416778 | 0.000609898 | AT-hook motif nuclear-localized protein 16-like [Dendrobium catenatum] | General Negative |
|  | TRINITY_DN18020_c0_g1 | 4.84 | 9.586666667 | up | 0.80560072 | 0.018479882 | protein PHOTOPERIOD-INDEPENDENT EARLY FLOWERING 1 [Dendrobium catenatum] | General Negative |
|  | TRINITY_DN922_c0_g3 | 5.63 | 12.1 | up | 0.953136541 | 0.01413171 | flowering time control protein FPA [Phalaenopsis equestris] | General Positive |
|  | TRINITY_DN15682_c0_g1 | 3.52 | 1.79 | down | -1.21 | 0.02 | FRIGIDA-like protein 2 [Phalaenopsis equestris] | Vernalization Negative |
|  | TRINITY_DN3166_c0_g1 | 3.32 | 2.08 | down | -0.96 | 0.02 | protein FD-like [Dendrobium catenatum] | Photoperiodism, light perception and signaling Positive |
|  | TRINITY_DN6550_c0_g2 | 44.63 | 83.81 | up | 0.65 | 0.00 | FT-interacting protein 1-like | Photoperiodism, light perception and signaling Positive |
|  | TRINITY_DN2646_c0_g1 | 11.38 | 8.39 | down | -0.71 | 0.04 | Zinc finger protein CONSTANS-LIKE 5 [Dendrobium catenatum] | Photoperiodism, light perception and signaling Positive |
|  | TRINITY_DN10875_c0_g1 | 9.336666667 | 4.633333333 | down | -1.167491959 | 0.006056306 | cyclic dof factor 3 [Dendrobium catenatum] | Photoperiodism, light perception and signaling Negative |
|  | TRINITY_DN497_c0_g3 | 8.153333333 | 5.023333333 | down | -0.887835734 | 0.016837135 | cyclic dof factor 1-like [Dendrobium catenatum] | Photoperiodism, light perception and signaling Negative |
|  | TRINITY_DN4435_c0_g1 | 27.11333333 | 42.49 | up | 0.699957847 | 0.010029253 | protein GIGANTEA [Dendrobium catenatum] | Photoperiodism, light perception and signaling Positive |
|  | TRINITY_DN8270_c0_g1 | 24.30333333 | 15.21666667 | down | -0.982220066 | 0.002201192 | squamosa promoter-binding-like protein 13 [Phalaenopsis equestris] | Aging Hormones Positive |
|  | TRINITY_DN9565_c0_g1 | 21.01666667 | 37.49 | up | 0.600936394 | 0.035495005 | squamosa promoter-binding-like protein 2 [Oncidium hybrid cultivar] | Aging Hormones Positive |
|  | TRINITY_DN633_c0_g1 | 10.44666667 | 6.633333333 | down | -1.047129264 | 0.016536657 | nuclear transcription factor Y subunit B-3-like [Phalaenopsis equestris] | Photoperiodism, light perception and signaling Positive |
|  | TRINITY_DN17234_c0_g2 | 0.21 | 0.97 | up | 2.02 | 0.02 | agamous-like 15 [Annona squamosa] | Photoperiodism, light perception and signaling Negative |
|  | TRINITY_DN6666_c0_g1 | 6.59 | 2.41 | down | -1.667018854 | 0.00252028 | flowering locus T [Agapanthus praecox subsp. orientalis] | Flowering time integrator Positive |
|  | TRINITY_DN2614_c1_g2 | 98.69 | 63.02333333 | down | -0.939232018 | 0.000215578 | MADS-box protein SVP isoform X1 [Dendrobium catenatum] | Flowering time integrator Negative |
|  | TRINITY_DN1394_c0_g1 | 0.95 | 2.456666667 | up | 1.447104687 | 0.005241428 | B-class MADS-box protein AP3-3, partial [Paphiopedilum hybrid cultivar] | Flower development and meristem identity |
|  | TRINITY_DN3646_c0_g2 | 0.89 | 0.466666667 | down | -1.116293007 | 0.038228351 | MADS-box transcription factor 23-like [Dendrobium catenatum] |  |
|  | TRINITY_DN3350_c0_g1 | 35.18 | 20.34666667 | down | -1.024782142 | 0.0000526172947918 | AP2-like ethylene-responsive transcription factor ANT [Dendrobium catenatum] |  |
|  | TRINITY_DN4425_c0_g1 | 19.49 | 12.66666667 | down | -0.857057522 | 0.001101715 | AP2-1 [Cymbidium goeringii] |  |
| stageⅥ | TRINITY_DN13667_c0_g1 | 70.25 | 29.01 | down | -1.329698331 | 4.10352E-06 | Ethylene-responsive transcription factor RAP2-7 [Apostasia shenzhenica] | Aging Photoperiodism, light perception and signaling Negative |
|  | TRINITY_DN8513_c0_g1 | 703.07 | 1568.2 | up | 1.096822117 | 1.52196E-06 | sucrose synthase [Bletilla striata] | Sugar Positive |
|  | TRINITY_DN8644_c0_g1 | 13.84 | 25.82 | up | 0.838342938 | 0.023423035 | squamosa promoter-binding-like protein 15 [Dendrobium catenatum] | Aging Positive |
|  | TRINITY_DN55824_c0_g1 | 1.51 | 0.513333333 | down | -1.653625524 | 0.006178298 | AT-hook motif nuclear-localized protein 22 | General NEGATIVE |
|  | TRINITY_DN15098_c0_g3 | 566.67 | 291.33 | down | -1.037166429 | 1.33624E-05 | FRIGIDA-like protein 4b [Dendrobium catenatum] | Vernalization NEGATIVE |
|  | TRINITY_DN3166_c0_g1 | 6.19 | 3.546666667 | down | -0.891069483 | 0.004357741 | protein FD-like [Dendrobium catenatum] | Photoperiodism, light perception and signaling Positive |
|  | TRINITY_DN41312_c0_g1 | 5.78 | 9.21 | up | 0.626637774 | 0.009795282 | FT-interacting protein 1 [Dendrobium catenatum] |  |
|  | TRINITY_DN3206_c0_g2 | 65.57 | 38.76333333 | down | -0.814953678 | 6.07094E-05 | cryptochrome-1 isoform X1 [Dendrobium catenatum] |  |
|  | TRINITY_DN633_c0_g2 | 8.18 | 13.72666667 | up | 0.70625445 | 0.008283789 | nuclear transcription factor Y subunit B-3-like [Phalaenopsis equestris] |  |
|  | TRINITY_DN11987_c0_g2 | 2.05 | 0.113333333 | down | -4.222728946 | 0.000403419 | MADS-box transcription factor 23 [Dendrobium catenatum] | Flower development and meristem identity |
|  | TRINITY_DN12247_c0_g1 | 43.36 | 120.45 | up | 1.406336099 | 9.43938E-06 | B-class MADS-box protein PI [Paphiopedilum hybrid cultivar] |  |
|  | TRINITY_DN1394_c0_g1 | 0.47 | 7.703333333 | up | 3.886367639 | 1.01854E-13 | B-class MADS-box protein AP3-3, partial [Paphiopedilum hybrid cultivar] |  |
|  | TRINITY_DN17292_c0_g1 | 3.21 | 0.14 | down | -3.94361902 | 4.78268E-06 | MADS-box transcription factor 27 [Apostasia shenzhenica] |  |
|  | TRINITY_DN2239_c0_g1 | 8.29 | 26.31666667 | up | 1.594674614 | 1.84982E-06 | MADS-box transcription factor 32 [Dendrobium catenatum] |  |
|  | TRINITY_DN2614_c1_g2 | 71.79 | 37.3 | down | -0.986015483 | 8.34053E-05 | MADS-box protein SVP isoform X1 [Dendrobium catenatum] |  |
|  | TRINITY_DN28035_c0_g2 | 0.35 | 3.36 | up | 3.245782494 | 0.002976955 | SEPALLATA-like MADS-box protein 1 [Paphiopedilum henryanum] |  |
|  | TRINITY_DN3676_c0_g1 | 3.72 | 5.846666667 | up | 0.601987762 | 0.03401428 | agamous-like MADS-box protein AGL80 [Dendrobium catenatum] |  |
|  | TRINITY_DN5540_c0_g1 | 8.29 | 1.34 | down | -2.703782384 | 0.008271888 | MADS-box transcription factor 26 [Elaeis guineensis] |  |
|  | TRINITY_DN1482_c2_g1 | 19.66 | 30.30666667 | up | 0.672238661 | 0.001528119 | Floral homeotic protein APETALA 2 [Apostasia shenzhenica] |  |
|  | TRINITY_DN6885_c0_g2 | 6.043333333 | 54.62 | up | 3.133496945 | 1.54368E-40 | TCP15 protein [Cymbidium ensifolium] | Bloting |
|  | TRINITY_DN2401_c2_g1 | 7.716666667 | 17.11333333 | up | 1.093348302 | 1.44984E-06 | TCP15 protein [Cymbidium ensifolium] |  |
|  | TRINITY_DN2852_c0_g1 | 523.4133333 | 1154.703333 | up | 1.084516991 | 8.98814E-07 | Xyloglucan endotransglucosylase/hydrolase protein 9 [Apostasia shenzhenica] |  |
|  | TRINITY_DN1947_c0_g1 | 2.503333333 | 8.846666667 | up | 1.789600963 | 1.94384E-07 | EPIDERMAL PATTERNING FACTOR-like protein 4 [Dendrobium catenatum] |  |
|  | TRINITY_DN3201_c0_g1 | 4.513333333 | 13.35333333 | up | 1.55025327 | 6.94503E-06 | EPIDERMAL PATTERNING FACTOR-like protein 4 [Dendrobium catenatum] |  |
|  | TRINITY_DN1481_c0_g1 | 4.473333333 | 9.66 | up | 1.061459069 | 6.58659E-05 | EPIDERMAL PATTERNING FACTOR-like protein 6 [Dendrobium catenatum] |  |
|  | TRINITY_DN1798_c0_g1 | 8.733333333 | 46.05333333 | up | 2.329377975 | 1.13772E-09 | growth-regulating factor 1-like isoform X1 [Phalaenopsis equestris] |  |
|  | TRINITY_DN8718_c0_g1 | 1.796666667 | 10.15333333 | up | 2.452552348 | 1.33562E-09 | growth-regulating factor 1-like [Phalaenopsis equestris] |  |
|  | TRINITY_DN9176_c0_g1 | 17.48666667 | 41.93333333 | up | 1.198102817 | 1.87651E-09 | growth-regulating factor 6 [Dendrobium catenatum] |  |
|  | TRINITY_DN8148_c0_g1 | 14.60333333 | 28.40333333 | up | 0.903357777 | 0.000324375 | growth-regulating factor 6 [Dendrobium catenatum] |  |
|  | TRINITY_DN991_c0_g1 | 30.73666667 | 55.88333333 | up | 0.840140078 | 0.001462465 | growth-regulating factor 4-like [Dendrobium catenatum] |  |
|  | TRINITY_DN10929_c0_g1 | 7.25 | 11.9 | up | 0.638640568 | 0.021491888 | growth-regulating factor 1-like isoform X1 [Phalaenopsis equestris] |  |

**Table S 6 Primers, Average Ct, SDS and coefficient of variation (CV) values used for the reference genes selection.**

| name | gene ID | forward primer(5'-3') | reward primer(5'-3') | NR description | Average Ct | SDs | CV |
| --- | --- | --- | --- | --- | --- | --- | --- |
| 60s | TRINITY_DN24966_c0_g2 | GGTGGGCATCTAGGGAGGTT | ATCCGCATTGGTCATCTTCG | 60S ribosomal protein L4-1 [Dendrobium catenatum] | 22.21444444 | 0.6566 | 0.0296 |
| EF2 | TRINITY_DN3773_c0_g1 | GAACTTGTAGGGAAGGCTCTG | TGATGGAGATGGAAGGTGAAAG | elongation factor 2 [Dendrobium catenatum] | 23.62076923 | 0.9583 | 0.0406 |
| HSP70 | TRINITY_DN9102_c0_g1 | CGACAAGCCGTTACCAATCC | CAACCCACGCATCCCCAT | heat shock 70 kDa protein, mitochondrial [Phalaenopsis equestris] | 27.45428571 | 1.6771 | 0.0611 |
| VHA-D | TRINITY_DN3922_c0_g1 | CGTTCGCTCGGCATCAGTA | GAGATCGGTGGAGGACTTGGA | V-type proton ATPase subunit D [Dendrobium catenatum] | 22.426 | 1.5091 | 0.0673 |
| ATP | TRINITY_DN22567_c0_g1 | CGCCTCCAGCAGCAACCC | TCAAAGCGGACATCAACCACA | ATP synthase subunit beta, mitochondrial [Vitis vinifera] | 28.34142857 | 1.5791 | 0.0557 |
| Acy1 | TRINITY_DN14267_c0_g3 | TGGGCGTTATTAGAGGTAGC | GGTGAAAATCCAAAAGCAGGTA | aminoacylase [Apostasia shenzhenica] | 25.31642857 | 1.38923115 | 0.055717641 |

**Table S 7 Primers used in the reference genes selection.**

| Name | Forward primer(5'-3') | Reverse primer(5'-3') | product length |
| --- | --- | --- | --- |
| MADS32 | CCCTCTGTTCCCAATGTTCA | CGTTCTCCTTGCCGTCCT | 100 |
| SEP | TCAGAATCCGTCACTCCGT | CCTGTAGTTCCTTGGTGGTTAG | 145 |
| MADS1 | GGCAATGGAAGGTAGTATGAGA | AAGTTGGGCTGGATAGGCT | 111 |
| XTH9 | TCAACGGAATCGGAAACC | CGAGAAAGACGACCTGCC | 108 |
| TCP15 | CCGTCCTTCATCGTTCCG | TGCTCCCACTGCCGTTTC | 136 |
| GA2OX | GGCTCCACTGCCCCTATG | AAGAATCCAAGCTCCTCGC | 133 |
| DAO | AGCTTCGTAACTCTCATCCAAG | TCCAAACCTTAGCCACATCG | 136 |
| CKX | CCCCACAACAGGTGAAATG | CAAAACTATGAAACCTTCCACA | 122 |
| DELLA | CGCCGAATCTATCGTCTCC | GCGGTGAAATGGGCAAAT | 119 |
| GID1 | TTCCAACGCTTACTATCATCG | TAACCACAGCACCATCGC | 142 |
| SAUR50 | AGCGGCGTTGAAGCAGAT | GCCCACATAAACGGCAAAG | 130 |
| SVP | CTTTGTGATGCTGAGGTGGG | TGGCTGGTCTGGTTTTGGT | 135 |
| RPL4 | GGTGGGCATCTAGGGAGGTT | ATCCGCATTGGTCATCTTCG | 138 |
